# Pelican Optimization Algorithm: A Novel Nature-Inspired Algorithm for Engineering Applications

**DOI:** 10.3390/s22030855

**Published:** 2022-01-23

**Authors:** Pavel Trojovský, Mohammad Dehghani

**Affiliations:** Department of Mathematics, Faculty of Science, University of Hradec Králové, 500 03 Hradec Králové, Czech Republic; mohammad.dehghani@uhk.cz

**Keywords:** optimization, nature inspired, swarm intelligence, optimization problem, pelican, population-based algorithm, stochastic

## Abstract

Optimization is an important and fundamental challenge to solve optimization problems in different scientific disciplines. In this paper, a new stochastic nature-inspired optimization algorithm called Pelican Optimization Algorithm (POA) is introduced. The main idea in designing the proposed POA is simulation of the natural behavior of pelicans during hunting. In POA, search agents are pelicans that search for food sources. The mathematical model of the POA is presented for use in solving optimization issues. The performance of POA is evaluated on twenty-three objective functions of different unimodal and multimodal types. The optimization results of unimodal functions show the high exploitation ability of POA to approach the optimal solution while the optimization results of multimodal functions indicate the high ability of POA exploration to find the main optimal area of the search space. Moreover, four engineering design issues are employed for estimating the efficacy of the POA in optimizing real-world applications. The findings of POA are compared with eight well-known metaheuristic algorithms to assess its competence in optimization. The simulation results and their analysis show that POA has a better and more competitive performance via striking a proportional balance between exploration and exploitation compared to eight competitor algorithms in providing optimal solutions for optimization problems.

## 1. Introduction

### 1.1. Motivation

Optimization is the study of selecting the optimum solution from a set of alternative solutions to a problem [1]. In fact, a problem that has more than one feasible solution is an optimization problem. Decision variables, constraints, and objective functions are the three main parts of each optimization problem for modeling [2]. From a general point of view, optimization problem-solving approaches are categorized into two groups: deterministic methods and stochastic methods [3]. Deterministic methods have difficulty solving complex optimization problems with discontinuous, high-dimensional, non-convex, and non-derivative objective functions. However, stochastic methods are able to overcome the difficulties of deterministic methods and provide appropriate solutions to optimization issues relying on random search in the problem-solving space and without using derivative and gradient information from the objective function of the optimization problem [4]. Population-based optimization algorithms are one of the efficient algorithms in the group of stochastic methods. These algorithms have been inspired by various phenomena of swarm intelligence, the natural behaviors of animals and insects, the laws of physics, the behavior of players and rules in various games, and the laws of evolution [5]. The process of finding the optimal solution in optimization algorithms is such that at first, a certain number of solvable solutions based on the constraints of the problem are generated randomly. These random solutions are then improved using the algorithm’s stages and a repetition-based procedure. The best recommended solution for the optimization issue is decided when the algorithm has been fully implemented. The basic optimum solution for an optimization issue is global optimal. However, the solutions provided by the optimization algorithms are not necessarily the same as the global optimal. Hence, the solution obtained by the optimization algorithms is called quasi-optimal [6]. The tendency to achieve better quasi-optimal solutions and closer to the global optimal has motivated researchers to develop countless optimization algorithms. Optimization algorithms are employed to achieve suitable solutions in various fields of science including image processing [7], sensor networks [8], engineering [9], and metric fixed-point applications [10,11].

### 1.2. Research Gap

Since countless optimization algorithms have been designed so far, the main question that arises is whether there is still a need to develop newer algorithms? The No Free Lunch (NFL) theorem answers this important question and challenge [12]. The NFL theorem illustrates the fact that an optimization algorithm may be highly capable of solving one set of optimization problems, but can fail to solve another set of optimization problems. This is due to the nature and different mathematical models of one real problem over another. Therefore, there is no guarantee that a particular optimization algorithm will be highly efficient in solving all optimization problems. The authors of this study were also motivated by the NFL Theorem to produce a novel optimization algorithm that can be employed to solve and prepare eligible quasi-optimal solutions to optimization issues.

### 1.3. Contribiution

The novelty and contribution of this research is in the development of a new optimization method named the Pelican Optimization Algorithm (POA), which is based on pelicans’ natural behaviors. The main idea in the design of POA is to model the behavior and strategy of pelicans during hunting. The various steps of the proposed POA are described and mathematically modeled. To test the effectiveness of the proposed POA in optimization, a set of twenty-three objective functions of unimodal and multimodal types have been used. In addition, the POA’s performance is compared with eight well-known optimization algorithms: Particle Swarm Optimization (PSO), Teaching–Learning-Based Optimization (TLBO), Gray Wolf Optimization (GWO), the Whale Optimization Algorithm (WOA), Marine Predators Algorithm (MPA), Tunicate Swarm Algorithm (TSA), Gravitational Search Algorithm (GSA), and the Genetic Algorithm (GA).

### 1.4. Paper Organization

The rest of the paper is organized in such a way that in Section 2, a study on optimization algorithms is presented. The proposed Pelican Optimization Algorithm (POA) is introduced in Section 3. Simulation studies are presented in Section 4. The discussion about the obtained results is provided in Section 5. The analysis of POA’s ability to solve engineering design problems is evaluated in Section 6. Finally, in Section 7, conclusions and recommendations for further research are stated.

## 2. Background

One of the most effective ways to tackle optimization issues is stochastic population-based optimization algorithms. Optimization algorithms in a general grouping based on the main ideas and inspiration used in their design can be grouped into four groups: swarm-based, evolutionary-based, physics-based, and game-based optimization algorithms.

Swarm-based optimization algorithms are developed with respect to natural phenomena: swarm behaviors of insects, animals, and other living things. Particle Swarm Optimization (PSO) is one of the oldest and most popular swarm-based algorithms inspired by the behavior of birds in search of food. In the PSO, the status of each population member is updated under the influence of the best position experienced by that member and the best position experienced by the total population [13]. Teaching–Learning-Based Optimization (TLBO) is developed from the simulation of a classroom atmosphere and the interactions between students and the teacher. In TLBO, population members are updated under teacher training and transfer their information to each other [14]. Gray Wolf Optimization (GWO) is inspired by the hierarchical structure and the social behavior of gray wolves when hunting. In GWO, four types of wolves, alpha, beta, delta, and omega, are used to model the hierarchical leadership of gray wolves, while population members are updated based on simulations of three main hunting stages, including the search for prey, encircling prey, and attacking prey [15]. A Whale Optimization Algorithm (WOA) is a nature-inspired, swarm-based optimization algorithm based on the modeling of humpback whale social behavior and their bubble-net hunting method. In WOA, population members are updated in three hunting phases including search for prey, encircling prey, and humpback whale bubble-net foraging behavior [16]. A Tunicate Swarm Algorithm (TSA) is developed based on simulation of jet propulsion and swarm behavior of tunicates during the navigation and foraging process. In TSA, the population is updated based on four phases including avoiding conflicts between search agents, moving towards the best neighbor, converging towards the best search agent, and swarm behavior [17]. Marine Predators Algorithm (MPA) is inspired by marine predators’ movement methods when capturing their prey in the seas. Because of the differing predator and prey speeds, the population update process in MPA has three phases: (i) predator be faster, (ii) the speed of the predator and the prey be equal, and (iii) prey be faster [18].

Evolutionary-based optimization algorithms are introduced based on simulations of biological sciences, genetic sciences, and other phenomena involving evolutionary processes. The Genetic Algorithm (GA) is one of the oldest and most widely used evolutionary algorithms, inspired by the reproductive process and Charles Darwin’s theory of natural selection. In GA, population members are updated based on three main operators: selection, crossover, and mutation [19]. The Artificial Immune System (AIS) algorithm is an evolutionary-based method derived from how the immune system works in the face of microbes and viruses. In AIS, the population update process is influenced by three phases: cognitive, activation, and effector [20].

Physics-based optimization algorithms are developed based on the modeling of the different laws of physics. Simulated Annealing is a physics-based algorithm inspired by the process of melting and cooling materials in metallurgy. In this physical process, the material is heated and gently cooled under controlled conditions to reduce its defects. Mathematical modeling of this process has been used in the design of the SA optimizer [21]. The Gravitational Search Algorithm (GSA) is inspired by the modeling of gravitational force between objects at different distances from each other. In the GSA, population members are updated based on the calculation of gravitational force and the modeling of Newtonian laws of motion [22].

Game-based optimization algorithms are designed based on simulating the rules of different individual and group games as well as the behavior of players in these games. The Football Game-based Optimizer (FGBO) is a game-based algorithm based on the simulation of player behavior and club interactions in the football game league. In FGBO, the population update process is based on the four phases of league holding, training, transfer of players between clubs, and promotion and relegation of clubs [23]. Tug of War Optimization (TWO) is based on simulating the behavior of players in a tug of war. In TWO, the process of updating population members is based on modeling the tensile force between members of the population who compete with each other [24].

Numerous optimization algorithms have been developed so far to solve optimization problems. To the best of our knowledge, however, there is no algorithm in the literature based on simulating the behavior and strategy of pelicans when hunting. The strategy of pelicans motivated the authors of this article to create a mathematical model of the social behavior of pelicans and to design a new optimization technique inspired by the hunting strategy of pelicans.

## 3. Pelican Optimization Algorithm

In this section, the inspiration and mathematical model of the proposed swarm-based Pelican Optimization Algorithm (POA) are presented.

### 3.1. Inspiration and Behavior of Pelican during Hunting

The pelican is large and has a long beak with a large bag in its throat that it uses to catch and swallow prey. This bird loves group and social life and lives in groups of several hundred pelicans [25]. The appearance of pelicans is as follows: they weigh about 2.75 to 15 kg, with a height of about 1.06 to 1.83 m, and a wingspan about 0.5 to 3 m [26]. Pelican food consists mainly of fish and more rarely of frogs, turtles, and crustaceans; if it is very hungry, it even eats seafood [27]. Pelicans often work together to hunt. The pelicans, after identifying the location of the prey, dive to their prey from a height of 10–20 m. Of course, some species also descend to their prey at lower altitudes. Then they spread their wings on the surface of the water to force the fish to go to shallow water so that they can catch their fish easily. When catching fish, a large amount of water enters the pelican’s beak, which moves the head forward before swallowing the fish to remove excess water [28].

The behavior and strategy of pelicans when hunting is an intelligent process that has made these birds skilled hunters. The main inspiration in the design of the proposed POA is originated from the modeling of the mentioned strategy.

### 3.2. Mathematical Model of the Proposed POA

The proposed POA is a population-based algorithm in which pelicans are members of this population. In population-based algorithms, each population member means a candidate solution. Each population member proposes values for the optimization problem variables according to their position in the search space. Initially, population members are randomly initialized according to the lower bound and upper bound of the problem using Equation (1).

(1) $$x_{i,j}=l_{j}+rand\cdot \left(u_{j}-l_{j}\right),\;i=1,2,\;\ldots ,\;N,\;\;j=1,2,\;\ldots ,m,$$

where $x_{i,j}$ is the value of the *j*th variable specified by the *i*th candidate solution, N is the number of population members, m is the number of problem variables, rand is a random number in interval [0, 1], $l_{j}$ is the *j*th lower bound, and $u_{j}$ is the *j*th upper bound of problem variables.

The population members of pelicans in the proposed POA are identified using a matrix called the population matrix in Equation (2). Each row of this matrix represents a candidate solution, while the columns of this matrix represent the proposed values for the problem variables.

(2) $$X={\left[\begin{array}{c} X_{1}\\ \vdots \\ X_{i}\\ \vdots \\ X_{N} \end{array}\right]}_{N\times m}={\left[\begin{array}{ccccc} x_{1,1} & \cdots & x_{1,j} & \cdots & x_{1,m}\\ \vdots & \ddots & \vdots & ⋰ & \vdots \\ x_{i,1} & \cdots & x_{i,j} & \cdots & x_{i,m}\\ \vdots & ⋰ & \vdots & \ddots & \vdots \\ x_{N,1} & \cdots & x_{N,j} & \cdots & x_{N,m} \end{array}\right]}_{N\times m},$$

where X is the population matrix of pelicans and $X_{i}$ is the *i*th pelican.

In the proposed POA, each population member is a pelican, which is a candidate solution to the given problem. Therefore, the objective function of the given problem can be evaluated based on each of the candidate solutions. The values obtained for the objective function are determined using a vector called the objective function vector in Equation (3).

(3) $$F={\left[\begin{array}{c} F_{1}\\ \vdots \\ F_{i}\\ \vdots \\ F_{N} \end{array}\right]}_{N\times 1}={\left[\begin{array}{c} F\left(X_{1}\right)\\ \vdots \\ F\left(X_{i}\right)\\ \vdots \\ F\left(X_{N}\right) \end{array}\right]}_{N\times 1},$$

where F is the objective function vector and $F_{i}$ is the objective function value of the *i*th candidate solution.

The proposed POA simulates the behavior and strategy of pelicans when attacking and hunting prey to update candidate solutions. This hunting strategy is simulated in two stages:

(i) Moving towards prey (exploration phase).
(ii) Winging on the water surface (exploitation phase).

#### 3.2.1. Phase 1: Moving towards Prey (Exploration Phase)

In the first phase, the pelicans identify the location of the prey and then move toward this identified area. Modeling this pelican’s strategy leads to search space scanning and the exploration power of the proposed POA in discovering different areas of search space. The important point in POA is that the location of the prey is generated randomly in the search space. This increases the exploration power of POA in the exact search of the problem-solving space. The above concepts and the pelican strategy in moving towards the place of prey are mathematically simulated in Equation (4).

(4) $$x_{i,j}^{P_{1}}=\{\begin{array}{cc} x_{i,j}+rand\cdot \left(p_{j}-I\cdot x_{i,j}\right), & F_{p}<F_{i};\\ x_{i,j}+rand\cdot \left(x_{i,j}-p_{j}\right), & \;\mathrm{else}, \end{array}$$

where $x_{i,j}^{P1}$ is the new status of the *i*th pelican in the *j*th dimension based on phase 1, I is a random number which is equal to one or two, $p_{j}$ is the location of prey in the *j*th dimension, and $F_{p}$ is its objective function value. The parameter *I* is a number that can be randomly equal to 1 or 2. This parameter is randomly selected for each iteration and for each member. When the value of this parameter is equal to two, it brings more displacement for a member, which can lead that member to newer areas of the search space. Therefore, parameter *I* affects the POA exploration power to accurately scan the search space.

In the proposed POA, the new position for a pelican is accepted if the value of the objective function is improved in that position. In this type of updating, which is called effective updating, the algorithm is prevented from moving to non-optimal areas. This process is modeled using Equation (5).

(5) $$X_{i}=\{\begin{array}{c} X_{i}^{P_{1}},\;\;F_{i}^{P_{1}}<F_{i};\\ X_{i},\;\;\;\;\;else, \end{array}$$

where $X_{i}^{P_{1}}$ is the new status of the *i*th pelican and $F_{i}^{P_{1}}$ is its objective function value based on phase 1.

#### 3.2.2. Phase 2: Winging on the Water Surface (Exploitation Phase)

In the second phase, after the pelicans reach the surface of the water, they spread their wings on the surface of the water to move the fish upwards, then collect the prey in their throat pouch. This strategy leads more fish in the attacked area to be caught by pelicans. Modeling this behavior of pelicans causes the proposed POA to converge to better points in the hunting area. This process increases the local search power and the exploitation ability of POA. From a mathematical point of view, the algorithm must examine the points in the neighborhood of the pelican location to converge to a better solution. This behavior of pelicans during hunting is mathematically simulated in Equation (6).

(6) $$x_{i,j}^{P_{2}}=x_{i,j}+R\cdot \left(1-\frac{t}{T}\right)\cdot \left(2\cdot rand-1\right)\cdot x_{i,j},\;$$

where $x_{i,j}^{P_{2}}$ is the new status of the *i*th pelican in the *j*th dimension based on phase 2, *R* is a constant, which is equal to 0.2, R·(1−t/T) is the neighborhood radius of $x_{i,j}$ while, t is the iteration counter, and T is the maximum number of iterations. The coefficient “R·(1−t/T)” represents the radius of the neighborhood of the population members to search locally near each member to converge to a better solution. This coefficient is effective on the POA exploitation power to get closer to the optimal global solution. In the initial iterations, the value of this coefficient is large and as a result, a larger area around each member is considered. As the algorithm replicates increases the “R·(1−t/T)” coefficient decreases, resulting in smaller radii of neighborhoods of each member. This allows us to scan the area around each member of the population with smaller and more accurate steps, so that the POA can converge to solutions closer to the global (and even exactly global) optimal based on the usage concept.

At this phase, effective updating has also been used to accept or reject the new pelican position, which is modeled in Equation (7).

(7) $$X_{i}=\{\begin{array}{c} X_{i}^{P_{2}},\;\;F_{i}^{P_{2}}<F_{i};\\ X_{i},\;\;\;\;\;else, \end{array}$$

where $X_{i}^{P_{2}}$ is the new status of the *i*th pelican and $F_{i}^{P_{2}}$ is its objective function value based on phase 2.

#### 3.2.3. Steps Repetition, Pseudo-Code, and Flowchart of the Proposed POA

After all population members have been updated based on the first and second phases, based on the new status of the population and the values of the objective function, the best candidate solution so far will be updated. The algorithm enters the next iteration and the different steps of the proposed POA based on Equations (4)–(7) are repeated until the end of the complete execution. Finally, the best candidate solution obtained during the algorithm iterations is presented as a quasi-optimal solution to the given problem.

**Figure 1:**
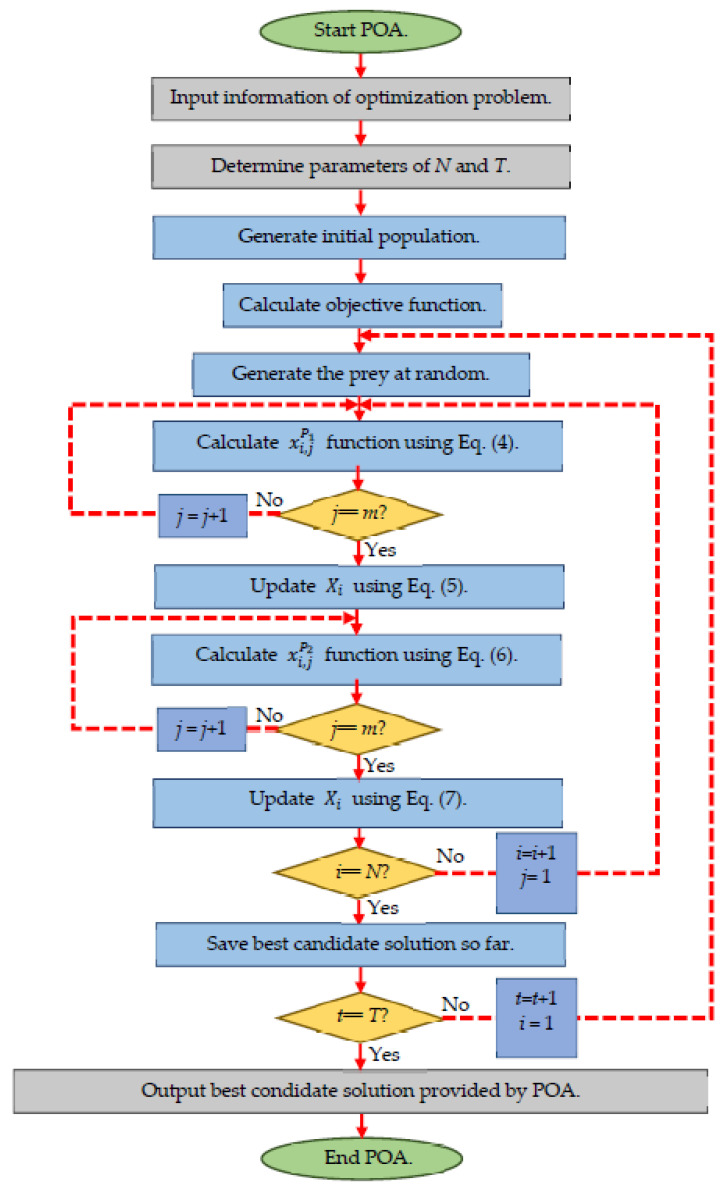
Flowchart of POA.

The various steps of the proposed POA are presented as a flowchart in Figure 1 and its pseudo-code in Algorithm 1.


**Algorithm 1.** Pseudo-code of POA.Start POA.1.Input the optimization problem information.2.Determine the POA population size (*N*) and the number of iterations (*T*).3.Initialization of the position of pelicans and calculate the objective function.4.For *t* = 1:*T*5. Generate the position of the prey at random.6. For *I* = 1:*N*7. Phase 1: Moving towards prey (exploration phase).8.  For *j* = 1:*m*9.   Calculate new status of the *j*th dimension using Equation (4).10.  End.11.  Update the *i*th population member using Equation (5).12. Phase 2: Winging on the water surface (exploitation phase).13.  For *j* = 1:*m.*14.   Calculate new status of the *j*th dimension using Equation (6).15.  End.16.  Update the *i*th population member using Equation (7).17. End.18. Update best candidate solution.19.End.20.Output best candidate solution obtained by POA.End POA.


### 3.3. Computational Complexity of the Proposed POA

In this subsection, the computational complexity of the proposed POA is calculated. The computational complexity of the proposed POA is based on four principles: algorithm initialization, evaluate the fitness function, generate prey, and solution updating. The computational complexity of the algorithm initialization processes is O(N). In each iteration, each population member evaluates the objective function in each of the two phases. So, the computational complexity of the fitness function evaluation is O(2·T·N). Given that prey is generated and evaluated at each iteration, O(T)+O(T·m) is the computational complexity of prey generation. In each iteration, the number of *N* population members that have *m* dimensions must be updated in two stages. Thus, the computational complexity of solutions updating is O(2·T·N·m). Therefore, the total computational complexity of the proposed POA is equal to O(N+T·(1+m)·(1+2·N)).

## 4. Simulation Studies and Results

In this section, the performance of the proposed POA in solving optimization problems is studied. For this purpose, POA is employed in solving twenty-three objective functions of different types of unimodal, high-dimensional multimodal, and fixed-dimensional multimodal. Details of the employed benchmark functions are specified in the Appendix A in Table A1, Table A2 and Table A3. In addition, the obtained optimization results from the proposed POA are compared with eight well-known optimization algorithms. These competing algorithms include (i) popular methods: Genetic Algorithm (GA) [19] and Particle Swarm Optimization (PSO) [13], (ii) popular and highly cited methods: Teaching–learning Based Optimization (TLBO) [14], Gray Wolf Optimization (GWO) [15], Whale Optimization Algorithm (WOA) [16], and Gravitational Search Algorithm (GSA) [22], and (iii) recently published methods: the Tunicate Swarm Algorithm (TSA) [17] and the Marine Predators Algorithm (MPA) [18]. Table 1 shows the values of the control parameters of these algorithms.

To evaluate the performance of the optimization algorithms, each of the competing algorithms, as well as the proposed POA in 20 independent implementations, each independent implementation containing 1000 iterations has been implemented on the objective functions. The simulation results are reported using four criteria: (i) the average of the best solutions obtained (avg), (ii) the standard deviation of the best solutions obtained (std), (iii) the best obtained candidate solution (bsf), and the median of the best solutions obtained (med). Two avg and std criteria are calculated using Equations (8) and (9).

(8) $$avg=\frac{1}{N_{r}}\cdot \sum_{i=1}^{N_{r}}BCS_{i},$$

(9) $$std=\sqrt{\frac{1}{N_{r}}\cdot \sum_{i=1}^{N_{r}}{\left(BCS_{i}-avg\right)}^{2}},$$

where $N_{r}$ is the number of independent implementations and $BCS_{i}$ is the best candidate solution obtained in the *i*th independent implementation for a given problem.

### 4.1. Evaluation of Unimodal Functions

The objective functions of F1 to F7 are of the unimodal type. The proposed POA and eight competitor algorithms are implemented on these functions. Table 2 shows the results of optimizing the F1 to F7 functions. According to this table, the proposed algorithm in the F6 optimization converges to the global optimal of this function, i.e., zero. In addition, the proposed POA is the first best optimizer in solving F1, F2, F3, F4, F5, and F7 functions. The POA has produced results that are significantly more competitive and closer to the global optimal than the rival algorithms, according to the comparison of the performance of optimization algorithms.

### 4.2. Evaluation of High-Dimensional Multimodal Functions

To analyze the proposed POA and eight competitor algorithms in optimizing high-dimensional multi-modal functions, six objective functions, F8 to F13, have been selected. Table 3 shows the results of the implementation of POA and eight competitor algorithms on these objective functions. The proposed POA presents the global optimal with convergence to zero for F9 and F11. The proposed algorithm is the first best optimizer in providing quasi-optimal solutions for F8 and F10. TLBO is the best optimizer for F12 while POA is the sixth-best optimizer in solving this objective function. GSA is also the best optimizer in solving F13. Analysis of the simulation results shows that the proposed POA has an acceptable ability to solve this type of optimization problems and is competitive with eight compared algorithms.

### 4.3. Evaluation of Fixed-Dimensional Multimodal Functions

F14 through F23 are ten objective functions that assess optimization algorithms’ capacity to tackle fixed-dimensional multimodal issues. The results of optimizing these objective functions using the proposed POA and eight competitor techniques are shown in Table 4. The proposed POA in optimizing F14 and F17 has been capable of converging to the global optimal of these functions. POA is the first best optimizer in solving F15, F19, F20 F21, F22, and F23. In optimizing the functions of F16, and F18 although the performance of the POA is similar to some competitor algorithms in the avg criterion, it has a better std criterion. Therefore, the proposed POA is more efficient to solve these objective functions. Analysis of the simulation results shows that the proposed POA has a higher ability to solve F14 to F23 fixed-dimensional multimodal optimization problems than the eight competitor algorithms.

**Figure 2:**
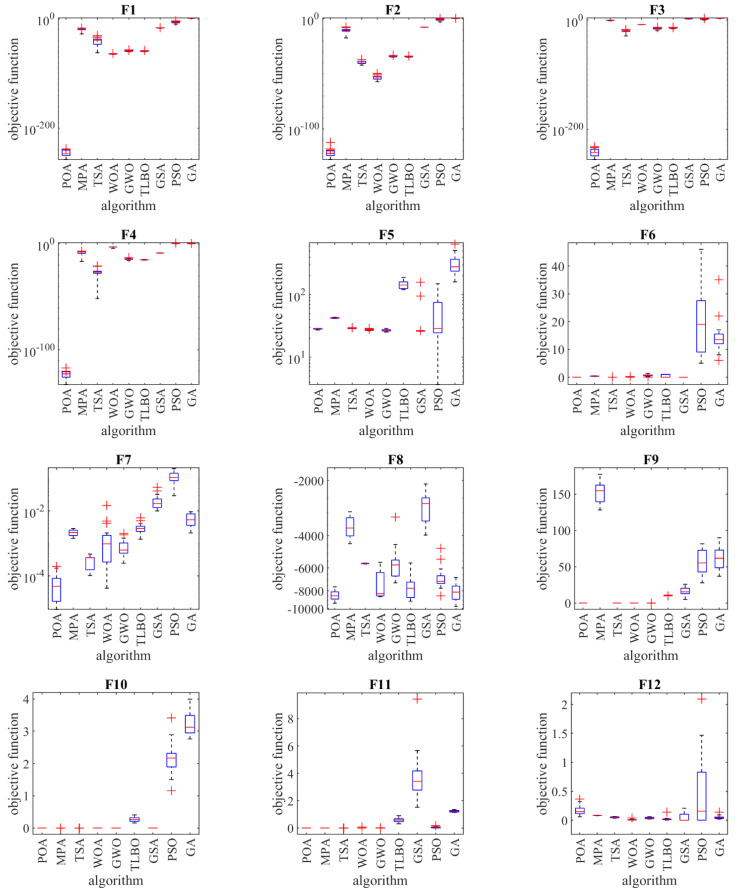

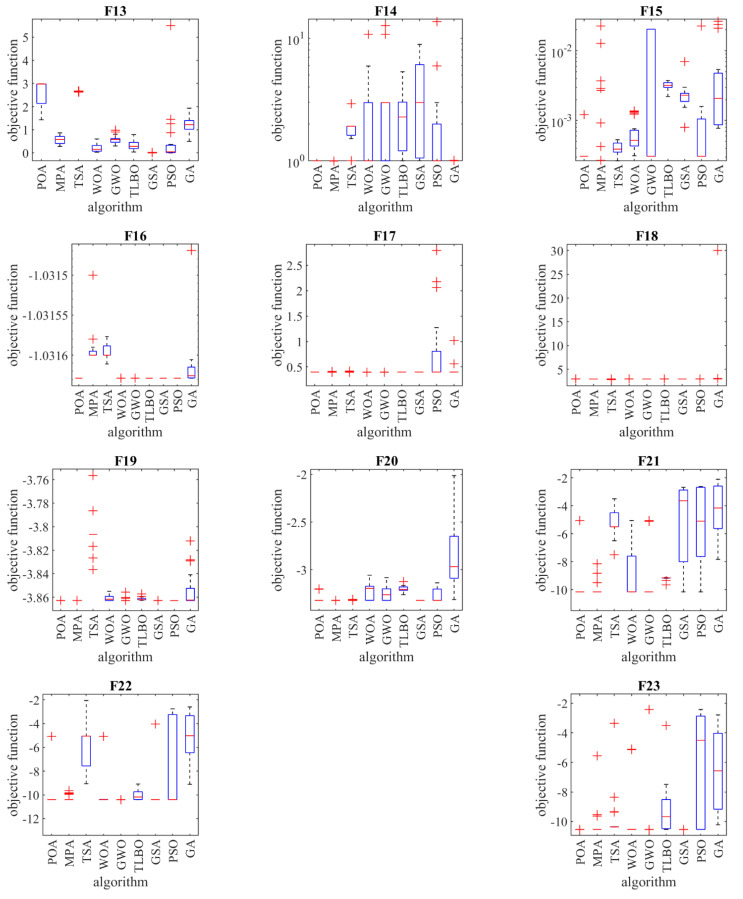
Boxplot of composition objective function results for different optimization algorithms.

The performance of the optimization algorithms and the proposed POA in solving the objective functions F1 to F23 are presented in Figure 2 as a boxplot.

### 4.4. Statistical Analysis

The use of average and std indices to report the optimization results of objective functions gives useful information about the comparison and performance of optimization techniques. Nevertheless, even after numerous separate executions, it is always conceivable that the superiority of one algorithm over several other algorithms be random. Therefore, in this subsection, a statistical analysis called a Wilcoxon sum rank test [29] is presented to show the superiority of the POA over eight competitor algorithms from a statistical point of view. The Wilcoxon sum rank test is a non-parametric statistical test that compares the similarity of two dependent samples. This test determines whether the difference between the two samples is statistically significant or not.

In the Wilcoxon sum rank test, an index called a *p*-value has been employed to determine the statistically significant difference between the performance of the two algorithms in optimizing different groups of objective functions. The simulation results of this test for the proposed POA with eight competitor algorithms are presented in Table 5. In this table, in cases where a p-value is less than 0.05, the proposed POA has a significant superiority over the competitor algorithm in that group of objective functions.

### 4.5. Sensitivity Analysis

The proposed POA is a population-based algorithm that converges to a quasi-optimal solution in an iterative process for a given optimization problem. Therefore, the values of these two parameters affect the performance of POA. In addition, the value of the parameter R in Equation (6) can also significantly affect the performance of the POA.

In this subsection, it has been studied the sensitivity analysis of the proposed POA with respect to the three parameters, namely the population number N, the maximum number of iterations T, and parameter R. There is not a general rule for setting values of N and T, but their values choice depends on factors such as the nature of the problem, the number of variables, constraints, and so on. Experimental knowledge and familiarity with the given optimization problem are very influential in choosing these two parameters. However, if there is no necessary knowledge and familiarity with the given problem, the values of these two parameters can be adjusted based on trial and error.

**Figure 3:**
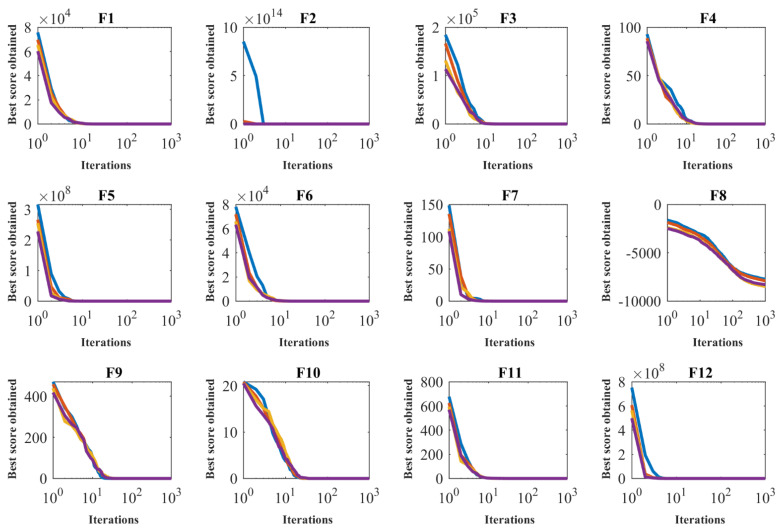

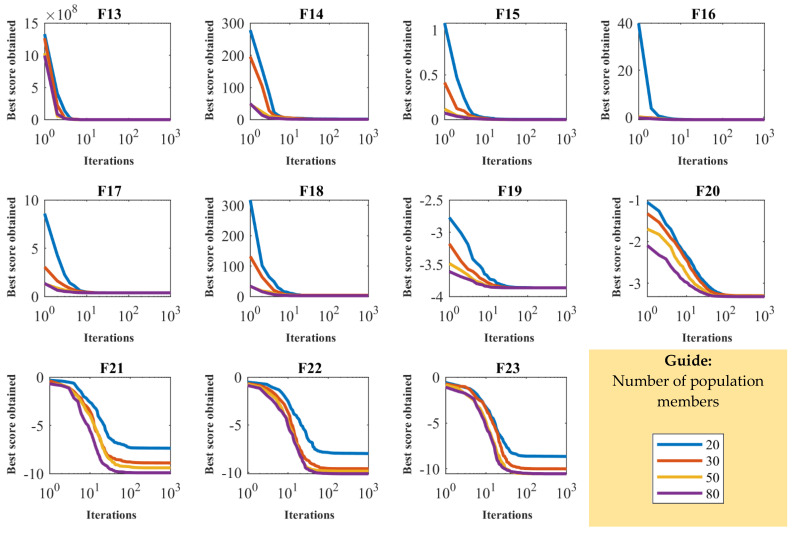
Sensitivity analysis of the POA to *N* parameter.

To evaluate the performance sensitivity of the proposed algorithm to the parameter N, POA for different populations of 20, 30, 50, and 80 members was implemented on the F1 to F23 objective functions. Table 6 shows the simulation results of the sensitivity analysis of the proposed POA to the parameter N. What can be deduced from this table is that the increase in population members has led to an increase in the exploratory power of the algorithm in search of search space and the discovery of more optimal areas. Therefore, as the number of population members increases, the value of the objective function decreases. Figure 3 shows the behavior of the convergence curves of the proposed POA in the sensitivity analysis to the parameter N.

**Figure 4:**
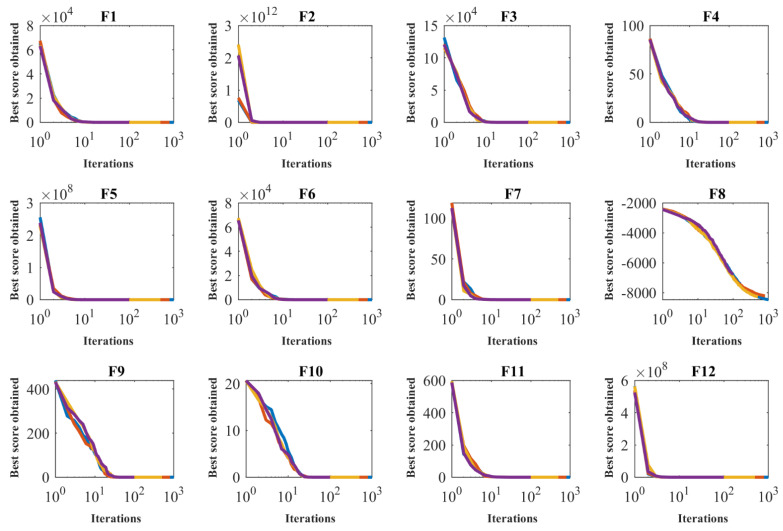

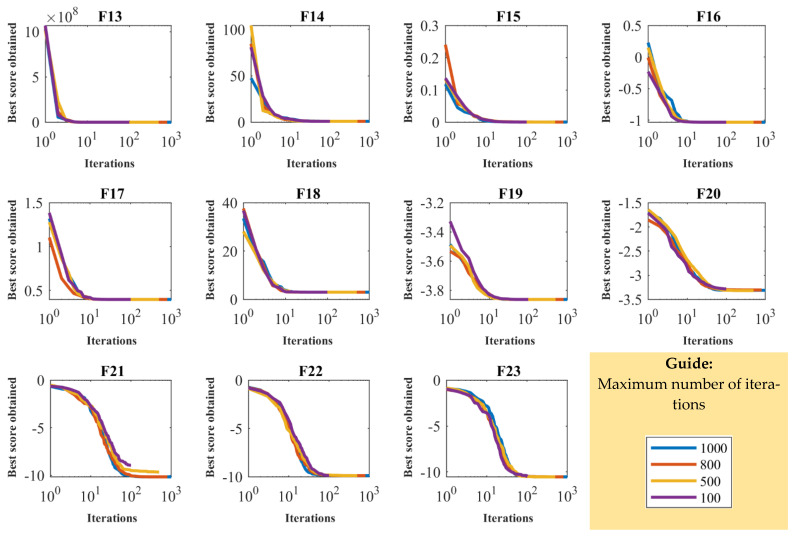
Sensitivity analysis of the POA to *T* parameter.

To analyze the sensitivity of the proposed algorithm to the parameter T, POA is applied to solve the objective functions F1 to F23 for the maximum number of iterations of 100, 500, 800, and 1000. The simulation results of the sensitivity of the proposed POA to the parameter T are presented in Table 7. Based on the results of this table, it was found that increasing the number of iterations of the algorithm gives more time to the population members to converge towards the optimal solution. Increasing the algorithm’s maximum number of iterations improves the algorithm’s exploitation power, allowing it to produce better solutions. The simulation results reveal that increasing the algorithm’s maximum number of iterations reduces the values of the goal functions. Figure 4 depicts the behavior of convergence curves under the effect of sensitivity analysis of the proposed POA to the *T*.

In order to analyze the sensitivity of POA to the parameter R, it should be noted that the coefficient “R·(1−t/T)” indicates that in each iteration, the maximum change for each member of the population is “R·(1−t/T)” times its current position. Therefore, the value of the parameter *R* in this coefficient must be less than one. The proposed POA for different values of R equal to 0.1, 0.2, 0.3, 0.4, 0.5, 0.6, 0.7, 0.8, 0.9, and 1 is employed in the optimization of the F1 to F23 function. The optimization results of F1 to F23 functions for different values of parameter R are reported in Table 8. The results of this sensitivity analysis show that the POA has a very low sensitivity to changes in the parameter R and in most cases provides the same solution. In optimizing of the functions F6, F9, F10, F11, F14, F15, F16, F17, F18, and F19, the different selected values for the parameter R had no effect on POA performance. In the general analysis and comparison of the results, it was found that POA has the best performance for the value of R equal to 0.2.

## 5. Discussion

Exploration power and exploitation power are two key and influential indicators of optimization algorithms’ success in obtaining solutions to optimization issues.

Exploitation power demonstrates the ability of the algorithm to search locally and converge as much as possible towards the global optimal. According to this concept, a good optimization algorithm should be able to accurately scan the space around the identified optimal area to provide a suitable quasi-optimal solution. Therefore it can be said that, compared to the performance of several optimization algorithms, the algorithm that converges to a better solution has a higher exploitation power. The F1 to F7 objective functions, which are of the unimodal type, have only one main peak and are therefore suitable for evaluating the exploitation power. The simulation results of these objective functions reported in Table 2 indicate the high exploitation ability of the proposed POA in local search and suitable convergence towards the global optimal. Analysis of the optimization results of these objective functions indicates very competitive and significant superiority of the proposed POA in the exploitation power and in providing a quasi-optimal solution over eight competing algorithms.

Exploration power demonstrates the ability of an algorithm to global search in problem-solving space and cross local optimal areas to discover the main optimal area. Accordingly, in comparing several optimization algorithms, an algorithm that scans the search space more accurately and is able to identify the area containing the global optimal has a higher exploration power. Exploration power is especially important in optimizing problems that have several local optimal in addition to the global optimal. F8 to 23 multimodal objective functions have this feature and are therefore suitable for evaluating the exploration power of optimization algorithms. The optimization results of these objective functions presented in Table 3 and Table 4 show that the proposed POA has a high exploration power for the global search in the problem-solving space and has been able to identify the optimal local area. Comparison and analysis of simulation results of F8 to F23 functions indicate the high exploration ability of POA compared to eight competing algorithms.

## 6. POA for Real-World Applications

In order to assess the effectiveness of POA in real-world purposes, this optimizer has been utilized to solve four engineering problems: pressure vessel design, speed reducer design, welded beam design, and tension/compression spring design.

### 6.1. Pressure Vessel Design

**Figure 5:**
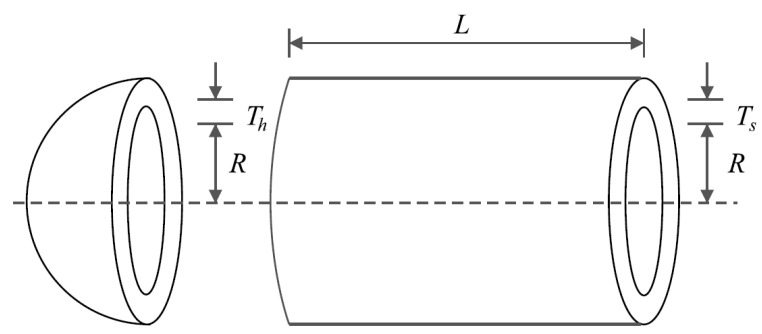
Schematic of pressure vessel design.

Pressure vessel design [30] is a minimization problem whose schematic is shown in Figure 5. The mathematical model of this problem is as follows:

*Consider* $X=\left[x_{1},\;x_{2},\;x_{3},\;x_{4}\right]=\left[T_{s},\;T_{h},\;R,\;L\right]$.

*Minimize* $f\;\left(x\right)=0.6224x_{1}x_{3}x_{4}+1.778x_{2}x_{3}^{2}+3.1661x_{1}^{2}x_{4}+19.84x_{1}^{2}x_{3}.$

*Subject to*:

$$g_{1}\;\left(x\right)=-x_{1}+0.0193x_{3}\;\leq \;0,$$

$$g_{2}\;\left(x\right)=-x_{2}+0.00954x_{3}\leq \;0,\;$$

$$g_{3}\;\left(x\right)=-\pi x_{3}^{2}x_{4}-\frac{4}{3}\pi x_{3}^{3}+1296000\leq \;0,\;$$

$$g_{4}\;\left(x\right)=x_{4}-240\;\leq \;0.\;$$

With

$$0\leq x_{1},x_{2}\leq 100,\;and\;10\leq x_{3},x_{4}\leq 200.$$

**Figure 6:**
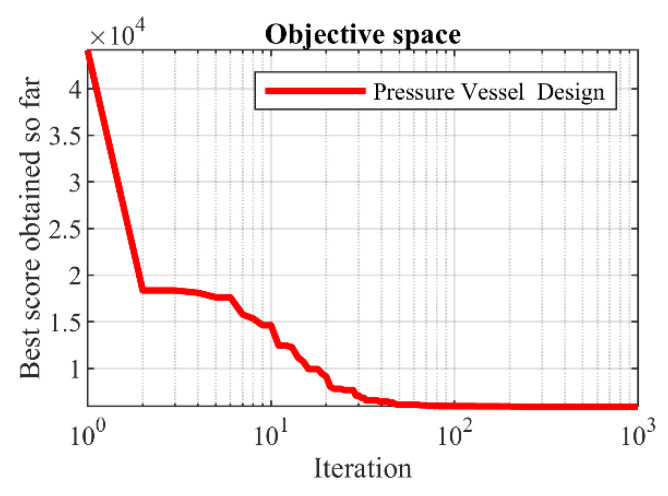
POA’s performance convergence curve on pressure vessel design.

The optimization results of this problem are presented in Table 9. POA provides the optimal solution with the values of the variables equal to (0.778035, 0.384607, 40.31261, and 199.9972) and the value of the objective function (5883.0278). The statistical results of the performance of competing and POA algorithms are reported in Table 10. Based on these results, POA, with better statistical indicators, has outperformed competing algorithms. Figure 6 shows the POA convergence curve in the pressure vessel design solution.

### 6.2. Speed Reducer Design Problem

**Figure 7:**
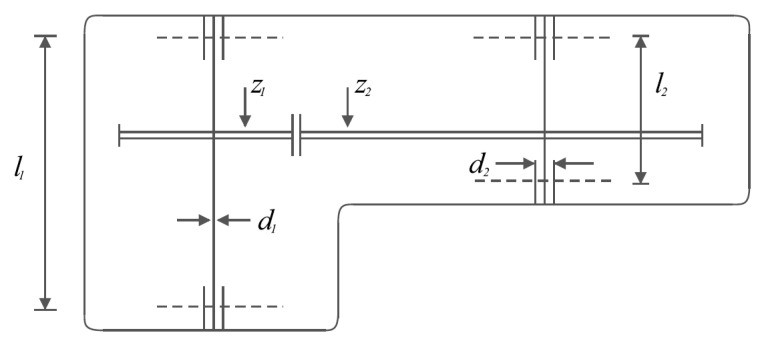
Schematic of speed reducer design.

Speed reducer design [31,32] is a weight minimization problem for a speed reducer. The schematic of this problem is shown in Figure 7 and the mathematical model of this problem is as follows:

*Consider*$X=\left[x_{1,}\;x_{2},\;x_{3},\;x_{4},\;x_{5},x_{6},x_{7}\right]=\left[b,\;m,\;p,\;l_{1},\;l_{2},\;d_{1},\;d_{2}\right]$.

*Minimize*$f\;\left(x\right)=0.7854x_{1}x_{2}^{2}\left(3.3333x_{3}^{2}+14.9334x_{3}-43.0934\right)-1.508x_{1}\left(x_{6}^{2}+x_{7}^{2}\right)+7.4777\left(x_{6}^{3}+x_{7}^{3}\right)+0.7854\left(x_{4}x_{6}^{2}+x_{5}x_{7}^{2}\right)$.

*Subject to:*

$$g_{1}\;\left(x\right)=\frac{27}{x_{1}x_{2}^{2}x_{3}}-1\;\leq \;0,\;$$

$$g_{2}\;\left(x\right)=\frac{397.5}{x_{1}x_{2}^{2}x_{3}}-1\leq \;0,\;$$

$$g_{3}\;\left(x\right)=\frac{1.93x_{4}^{3}}{x_{2}x_{3}x_{6}^{4}}-1\leq \;0,\;$$

$$g_{4}\;\left(x\right)=\frac{1.93x_{5}^{3}}{x_{2}x_{3}x_{7}^{4}}-1\;\leq \;0,\;$$

$$g_{5}\left(x\right)=\frac{1}{110x_{6}^{3}}\sqrt{{\left(\frac{745x_{4}}{x_{2}x_{3}}\right)}^{2}+16.9\times 10^{6}}-1\leq \;0,\;$$

$$g_{6}\left(x\right)=\frac{1}{85x_{7}^{3}}\sqrt{{\left(\frac{745x_{5}}{x_{2}x_{3}}\right)}^{2}+157.5\times 10^{6}}-1\;\leq \;0,\;$$

$$g_{7}\;\left(x\right)=\frac{x_{2}x_{3}}{40}-1\;\leq \;0,\;$$

$$g_{8}\;\left(x\right)=\frac{5x_{2}}{x_{1}}-1\;\leq \;0,\;$$

$$g_{9}\;\left(x\right)=\frac{x_{1}}{12x_{2}}-1\;\leq \;0,\;$$

$$g_{10}\;\left(x\right)=\frac{1.5x_{6}+1.9}{x_{4}}-1\;\leq \;0,\;$$

$$g_{11}\;\left(x\right)=\frac{1.1x_{7}+1.9}{x_{5}}-1\;\leq \;0.$$

With

$$2.6\leq x_{1}\leq 3.6,\;0.7\leq x_{2}\leq 0.8,\;17\leq x_{3}\leq 28,\;7.3\leq x_{4}\leq 8.3,\;7.8\leq x_{5}\leq 8.3,\;2.9\leq x_{6}\leq 3.9,\;and\;5\leq x_{7}\leq 5.5.$$

**Figure 8:**
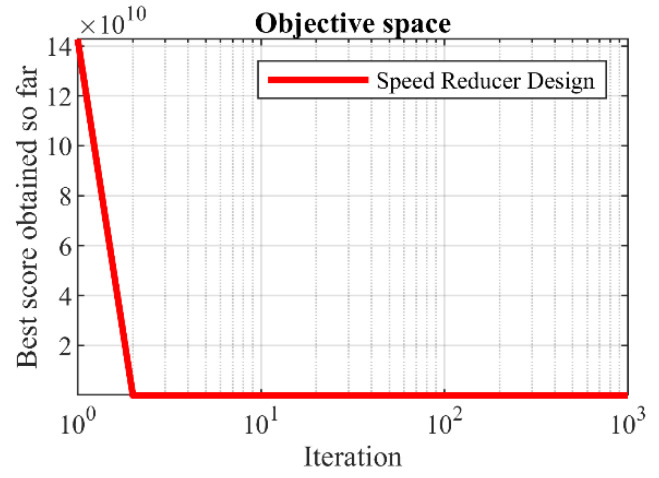
POA’s performance convergence curve on speed reducer design.

The values obtained from different algorithms are reported in Table 11. Based on these results, it is clear that POA has provided the optimal solution to this problem with the values of the variables equal to (3.5, 0.7, 17, 7.3, 7.88, 3.350215, and 5.286683) and the value of the objective function (2996.3482). The statistical results obtained from the implementation of competitor algorithms and POA on the speed reducer design problem are presented in Table 12. The analysis of these results indicates the superiority of POA in the effective solution of this problem due to having better values for statistical indicators. The POA convergence curve during speed reducer design optimization is shown in Figure 8.

### 6.3. Welded Beam Design

**Figure 9:**
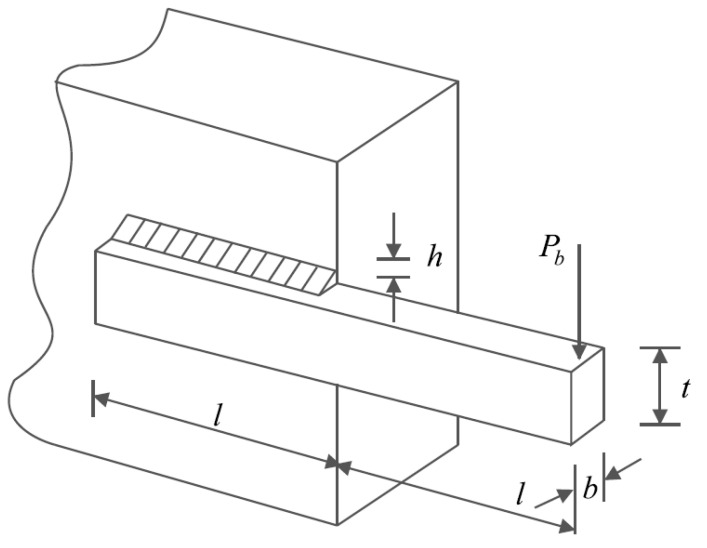
Schematic of welded beam design.

Welded beam design [16] is a minimizing problem of the fabrication cost of welded beam which the schematic of this problem is shown in Figure 9. The mathematical model of this problem is as follows:

*Consider*$X=\left[x_{1},\;x_{2},\;x_{3},\;x_{4}\right]=\left[h,\;l,\;t,\;b\right]$.

*Minimize*$f\;\left(x\right)=1.10471x_{1}^{2}x_{2}+0.04811x_{3}x_{4}\;\left(14.0+x_{2}\right)$.

*Subject to:*

$$g_{1}\;\left(x\right)=\tau \left(x\right)-13600\;\leq \;0,\;$$

$$g_{2}\;\left(x\right)=\sigma \left(x\right)-30000\;\leq \;0,\;$$

$$g_{3}\;\left(x\right)=x_{1}-x_{4}\leq \;0,\;$$

$$g_{4}\;\left(x\right)=0.10471x_{1}^{2}+0.04811x_{3}x_{4}\;\left(14+x_{2}\right)-5.0\;\leq \;0,\;$$

$$g_{5}\left(x\right)=0.125-x_{1}\leq \;0,\;$$

$$g_{6}\left(x\right)=\delta \;\left(x\right)-0.25\;\leq \;0,\;$$

$$g_{7}\;\left(x\right)=6000-p_{c}\;\left(x\right)\;\leq \;0.\;$$

*Where*

$$\tau \left(x\right)=\sqrt{\tau^{\prime }+\left(2\tau \tau^{\prime }\right)\frac{x_{2}}{2R}+{\left(\tau \prime \prime \right)}^{2}},\;$$

$$\tau^{\prime }=\frac{6000}{\sqrt{2}x_{1}x_{2}},\;$$

$$\tau \prime \prime =\frac{MR}{J},\;$$

$$M=6000\left(14+\frac{x_{2}}{2}\right),\;$$

$$R=\sqrt{\frac{x_{2}^{2}}{4}+{\left(\frac{x_{1}+x_{3}}{2}\right)}^{2}},\;$$

$$J=2\left\{x_{1}x_{2}\sqrt{2}\left[\frac{x_{2}^{2}}{12}+{\left(\frac{x_{1}+x_{3}}{2}\right)}^{2}\right]\right\},\;$$

$$\sigma \left(x\right)=\frac{504000}{x_{4}x_{3}^{2}}\;$$

$$\delta \;\left(x\right)=\frac{65856000}{\left(30\cdot 10^{6}\right)x_{4}x_{3}^{3}},\;$$

$$p_{c}\;\left(x\right)=\frac{4.013\left(30\cdot 10^{6}\right)\sqrt{\frac{x_{3}^{2}x_{4}^{6}}{36}}}{196}\left(1-\frac{x_{3}}{28}\sqrt{\frac{30\cdot 10^{6}}{4\left(12\cdot 10^{6}\right)}}\right).\;$$

With

$$0.1\leq x_{1},\;x_{4}\leq 2\;and\;0.1\leq x_{2},\;x_{3}\leq 10.$$

**Figure 10:**
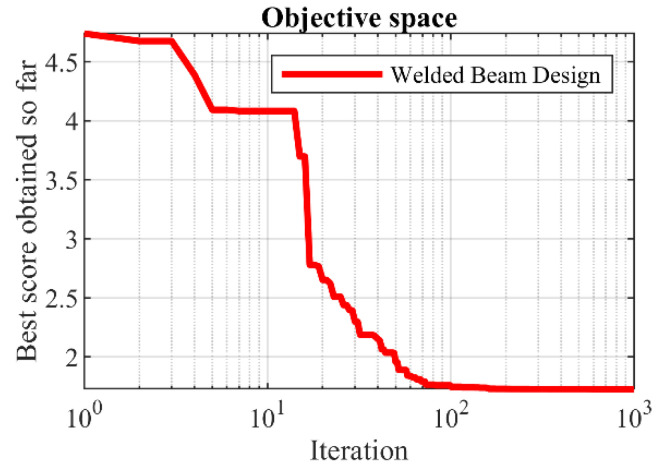
POA’s performance convergence curve on welded beam design.

The optimization results of the welded beam design problem are presented in Table 13. The POA provides the optimal solution to this problem by assigning the values of the variables equal to (0.205719, 3.470104, 9.038353, and 0.205722) and the value of the objective function (1.725021). The statistical results of performance of POA and eight competitor algorithms in optimizing this problem are reported in Table 14. Comparison of the results shows that POA has the superior ability to introduce optimal values of variables over eight competitor algorithms. The POA convergence curve to the optimal solution of the welded beam design problem is shown in Figure 10.

### 6.4. Tension/Compression Spring Design Problem

**Figure 11:**
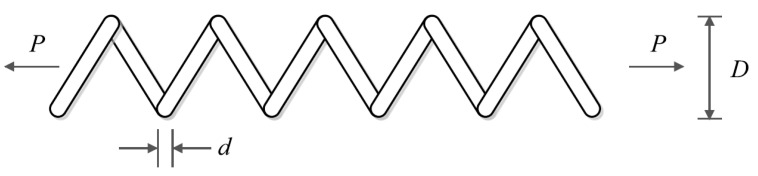
Schematic of tension/compression spring design.

Tension/compression spring design [16] is a weight minimization problem, a schematic of which is shown in Figure 11. The mathematical model of this problem is as follows:

*Consider* $X=\left[x_{1},\;x_{2},\;x_{3}\;\right]=\left[d,\;D,\;P\right].$

*Minimize* $f\;\left(x\right)=\left(x_{3}+2\right)x_{2}x_{1}^{2}.$

*Subject to:*

$$g_{1}\;\left(x\right)=1-\frac{x_{2}^{3}x_{3}}{71785x_{1}^{4}}\;\leq \;0,\;$$

$$g_{2}\;\left(x\right)=\frac{4x_{2}^{2}-x_{1}x_{2}}{12566\left(x_{2}x_{1}^{3}\right)}+\frac{1}{5108x_{1}^{2}}-1\leq \;0,\;$$

$$g_{3}\;\left(x\right)=1-\frac{140.45x_{1}}{x_{2}^{2}x_{3}}\leq \;0,$$

$$g_{4}\;\left(x\right)=\frac{x_{1}+x_{2}}{1.5}-1\;\leq \;0.$$

With

$$0.05\leq x_{1}\leq 2,\;0.25\leq x_{2}\leq 1.3\;and\;2\leq \;x_{3}\leq 15.$$

**Figure 12:**
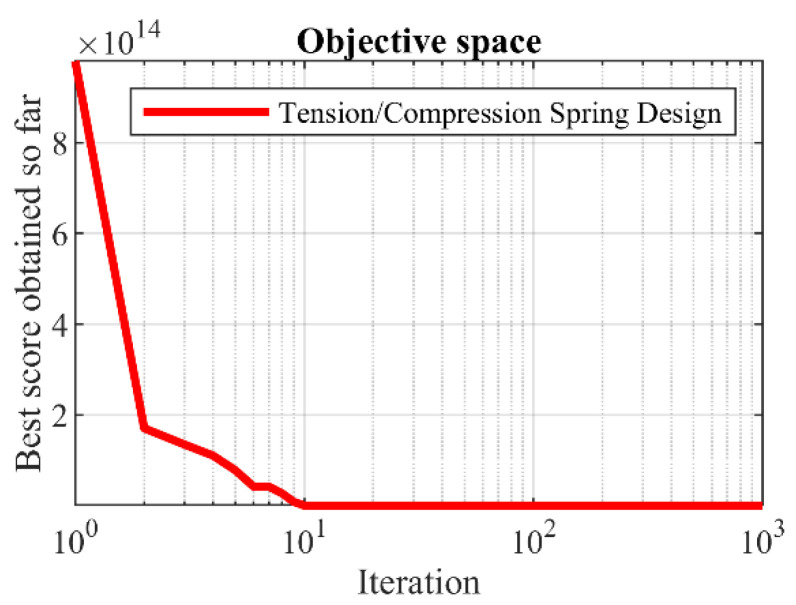
POA’s performance convergence curve on tension/compression spring.

The implementation results of POA and eight competitor algorithms on solving tension/compression spring design are reported in Table 15. Based on the results, it is clear that POA has provided the optimal solution by presenting the values of the variables equal to (0.051892, 0.361608, and 11.00793) and the value of the objective function (0.012666). The statistical results of the performance of the employed algorithms are presented in Table 16. What can be deduced from this table is that POA is a more effective optimizer for solving the tension/compression spring design problem against eight competitor algorithms by providing better statistical indicators. The convergence curve of POA during convergence to the optimal solution of this problem is shown in Figure 12.

### 6.5. The POA’s Applicability in Image Processing and Sensor Networks

Interconnected sensors collect huge amounts of data which are frequently useful in a variety of contexts. In today’s digital transformation era, numerous sorts of sensors and networks reinforce the usage of artificial intelligence and big data science. These data are primarily unstructured and well specified within the context of artificial intelligence, machine learning, data science, and big data. Data from medical images, traceability of infected patients, environmental monitoring, mobility in public transport, etc., usually georeferenced, are very interesting for the above research. These huge amounts of data come from a variety of sources, ranging from social media to IoT sensors. For these sorts of observations, classical methods for structured data analysis are insufficient and inadequate for discovering relevant knowledge and obtaining information. As a result, artificial intelligence approaches, such as the use of the proposed POA for various applications in sensor and image processing networks, are becoming increasingly important. In general, the application of the proposed POA in solving optimization problems might enable a broad range of future tasks in image processing, wireless sensor networks, signal denoising, machine learning, power systems, artificial intelligence, big data, COVID-19 modeling, data mining, feature selection, and other benchmark functions.

## 7. Conclusions and Future Works

In this paper, a new swarm-based optimization algorithm called the Pelican Optimization Algorithm (POA) was presented. The fundamental inspiration of the proposed POA is the strategy and behavior of pelicans during hunting. These behaviors include diving towards their prey and fluttering wings on the surface of the water. The various steps of POA were described and then its mathematical modeling was presented for use in solving optimization problems. The proposed algorithm was tested by solving twenty-three objective functions belonging to unimodal, high-dimensional multimodal, and fixed-dimensional multimodal. Additionally, to further analyze the capabilities of the proposed algorithm, the optimization results obtained from POA are compared with the performance of eight well-known algorithms, including WOA, TSA, GWO, MPA, GSA, GA, TLBO, and PSO. The optimization results of unimodal functions indicated the high exploitation power of the proposed POA in converging towards the global optimal solution. The simulation results of these functions showed that POA has significant superiority over eight competitor algorithms in solving unimodal problems. The simulation outcomes of multimodal functions demonstrated the suggested POA’s high exploration power in effective checking of the search space and finding of the optimal area. The simulation results demonstrated that the POA approach outperformed eight competitor algorithms in handling multimodal optimization issues. Based on the simulation results, it is possible to infer that the suggested POA is highly efficient in addressing optimization issues and is far more competitive and superior to similar methods. In addition, POA was employed for solving four engineering design problems, including pressure vessel design, speed reducer design, welded beam design, and tension/compression spring design. The simulation results showed that POA has a satisfactory performance in effectively solving design problems in real-world applications.

The authors provide several research directions for future studies with respect to this paper. Among the specific research potentials of the proposed method are the development of binary and multi-objective versions of POA. Furthermore, the authors’ ideas for future research include the application of the POA in tackling optimization issues in various science fields and real-world challenges. Note that the proposed POA might enable a broad range of future tasks. This includes applying this algorithm in numerous applications such as, e.g., image processing, wireless sensor networks, signal denoising, machine learning, power systems, artificial intelligence, big data, COVID-19 modeling, data mining, feature selection, and other benchmark functions. Like all stochastic optimization techniques, one of the limitations of the proposed POA is that new optimizers may be developed in the future that will perform better than POA in some real applications. Additionally, due to the stochastic nature of the POA solution method, it cannot be guaranteed that the solutions obtained using POA for optimization problems are exactly equal to the global optimum for all optimization problems.

## Figures and Tables

**Table 1:** Parameter values for the compared algorithms.

Algorithm	Parameter	Value
MPA	Binary vector	*U* = 0 or 1
	Random vector	R is a vector of uniform random numbers in [0, 1].
	Constant number	*p* = 0.5
	Fish Aggregating Devices (FADs)	*FADs* = 0.2
TSA	c1, c2, c3	random numbers lie in the interval [0, 1].
	Pmin	1
	Pmax	4
WOA	l is a random number in [−1, 1].	
	ris a random vector in [0, 1].	
	Convergence parameter (a)	a: Linear reduction from 2 to 0.
GWO	Convergence parameter (a)	a: Linear reduction from 2 to 0.
TLBO	random number	rand is a random number from interval [0, 1].
	*T_F_*: teaching factor	$T_{F}=\mathrm{round}\;\left[\left(1+rand\right)\right]$
GSA	Alpha	20
	G_0_	100
	Rnorm	2
	Rnorm	1
PSO	Velocity limit	10% of dimension range
	Topology	Fully connected
	Inertia weight	Linear reduction from 0.9 to 0.1
	Cognitive and social constant	$\left(C_{1},\;C_{2}\right)=\left(2,\;2\right)$
GA	Type	Real coded
	Mutation	Gaussian (Probability = 0.05)
	Crossover	Whole arithmetic (Probability = 0.8)
	Selection	Roulette wheel (Proportionate)

**Table 2:** Evaluation results of unimodal functions.

		GA	PSO	GSA	TLBO	GWO	WOA	TSA	MPA	POA
F_1_	avg	11.6208	4.1728 × 10^−4^	2.0259 × 10^−16^	3.8324 × 10^−59^	1.0896 × 10^−57^	5.37 × 10^−62^	5.7463 × 10^−37^	3.2612 × 10^−20^	2.87 × 10^−258^
	std	2.6142 × 10^−11^	3.6142 × 10^−21^	6.9113 × 10^−30^	9.6318 × 10^−72^	5.1462 × 10^−73^	5.78 × 10^−78^	6.3279 × 10^−20^	1.5264 × 10^−19^	4.51 × 10^−514^
	bsf	5.593489	2 × 10^−10^	8.2 × 10^−18^	9.36 × 10^−61^	7.73 × 10^−61^	1.61 × 10^−65^	1.14 × 10^−62^	3.41 × 10^−28^	7.62 × 10^−264^
	med	11.04546	9.92 × 10^−7^	1.78 × 10^−17^	4.69 × 10^−60^	1.08 × 10^−59^	8.42 × 10^−54^	3.89 × 10^−38^	1.27 × 10^−19^	8.2 × 10^−248^
F_2_	avg	4.6942	0.3114	7.0605 × 10^−7^	4.6237 × 10^−34^	2.0509 × 10^−33^	2.51 × 10^−55^	4.5261 × 10^−38^	6.3214 × 10^−11^	1.43× 10^−128^
	std	5.4318 × 10^−14^	4.4667 × 10^−16^	8.5637 × 10^−23^	9.3719 × 10^−49^	6.3195 × 10^−29^	5.60 × 10^−58^	2.6591 × 10^−40^	3.6249 × 10^−11^	2.90× 10^−129^
	bsf	1.591137	0.001741	1.59 × 10^−8^	1.32 × 10^−35^	1.55 × 10^−35^	3.42 × 10^−63^	8.26 × 10^−43^	4.25 × 10^−18^	2.61 × 10^−131^
	med	2.463873	0.130114	2.33 × 10^−8^	4.37 × 10^−35^	6.38 × 10^−35^	1.59 × 10^−51^	8.26 × 10^−41^	3.18 × 10^−11^	7.1 × 10^−123^
F_3_	avg	1361.2743	588.3012	280.6014	7.0772 × 10^−14^	4.7206 × 10^−14^	7.5621 × 10^−9^	5.6230 × 10^−20^	0.0819	1.88× 10^−256^
	std	6.6096 × 10^−12^	9.7117 × 10^−12^	5.2497 × 10^−12^	8.9637 × 10^−30^	6.5225 × 10^−28^	1.02 × 10^−18^	7.0925 × 10^−19^	0.1370	5.16× 10^−614^
	bsf	1014.689	1.614937	81.91242	1.21 × 10^−16^	4.75 × 10^−20^	1.9738 × 10^−11^	7.29 × 10^−30^	0.032038	7.36 × 10^−262^
	med	1510.715	54.15445	291.4308	1.86 × 10^−15^	1.59 × 10^−16^	17085.2	9.81 × 10^−21^	0.378658	8.2 × 10^−244^
F_4_	avg	2.0396	4.3693	2.6319 × 10^−8^	8.9196 × 10^−14^	1.9925 × 10^−13^	0.0013	3.1162 × 10^−22^	6.3149 × 10^−8^	2.36× 10^−133^
	std	4.3321× 10^−14^	4.2019 × 10^−15^	5.3017 × 10^−23^	1.7962 × 10^−29^	1.8305 × 10^−28^	0.0877	6.3129 × 10^−21^	2.3687 × 10^−9^	8.37× 10^−134^
	bsf	1.389849	1.60441	2.09 × 10^−09^	6.41 × 10^−16^	3.43 × 10^−16^	0.0001	1.87 × 10^−52^	3.42 × 10^−17^	6.08 × 10^−138^
	med	2.09854	3.260672	3.34 × 10^−09^	1.54 × 10^−15^	7.3 × 10^−15^	0.0010	3.13 × 10^−27^	3.03 × 10^−08^	2.8 × 10^−123^
F_5_	avg	308.4196	50.5412	36.01528	147.6214	27.1786	27.17543	28.8592	46.0408	27.1253
	std	3.0412 × 10^−12^	1.8529 × 10^−13^	2.6091 × 10^−13^	6.3017 × 10^−13^	8.7029 × 10^−14^	0.393959	4.3219 × 10^−3^	0.4199	1.91× 10^−15^
	bsf	160.5013	3.647051	25.83811	120.7932	25.21201	26.43249	28.53831	41.58682	26.2052
	med	279.5174	28.69298	26.07475	142.8936	26.70874	26.93542	28.53913	42.49068	28.707
F_6_	avg	15.6231	20.2691	0	0.5531	0.6518	0.071527	5.7268 × 10^−20^	0.3894	0
	std	7.3160 × 10^−14^	2.6314	0	3.1971 × 10^−15^	5.3096 × 10^−16^	0.006113	2.1163 × 10^−24^	0.2001	0
	bsf	6	5	0	0	1.57 × 10^−05^	0.014645	6.74 × 10^−26^	0.274582	0
	med	13.5	19	0	0	0.621487	0.029296	6.74 × 10^−21^	0.406648	0
F_7_	avg	8.6517 × 10^−2^	0.3218	0.0234	0.0011	0.0077	0.00103	8.2196 × 10^−4^	1.2561× 10^−3^	9.37× 10^−6^
	std	8.9206 × 10^−17^	3.4333 × 10^−16^	7.1526 × 10^−17^	3.2610 × 10^−18^	7.2307 × 10^−19^	1.12 × 10^−5^	9.6304 × 10^−5^	9.6802× 10^−3^	8.03× 10^−20^
	bsf	0.002111	0.029593	0.01006	0.001362	0.000248	4.24 × 10^−5^	0.000104	0.001429	7.05 × 10^−07^
	med	0.005365	0.107872	0.016995	0.002912	0.000629	0.00215	0.000367	0.00218	4.86 × 10^−05^

**Table 3:** Evaluation results of high-dimensional multimodal functions.

		GA	PSO	GSA	TLBO	GWO	WOA	TSA	MPA	POA
F_8_	avg	−8210.3415	−6899.9556	−2854.5207	−7410.8016	−5903.3711	−7239.1	−5737.7822	−3611.2271	−9336.7304
	std	833.5126	625.4286	2641576	513.4752	467.8216	261.0117	39.5203	811.1459	2.64× 10^−12^
	bsf	−9717.68	−8501.44	−3969.23	−9103.77	−7227.05	−7568.9	−5706.3	−4419.9	−9850.21
	med	−8117.66	−7098.95	−2671.33	−7735.22	−5774.63	−7124.8	−5669.63	−3632.84	−8505.55
F_9_	avg	62.1441	57.0503	16.5714	10.1379	8.1036 × 10^−14^	0	6.0311 × 10^−3^	139.9806	0
	std	2.1637 × 10^−13^	6.0013 × 10^−14^	6.1972 × 10^−14^	4.9631 × 10^−14^	4.6537 × 10^−29^	0	5.6146 × 10^−3^	25.9024	0
	bsf	36.86623	27.85883	4.974795	9.873963	0	0	0.004776	128.2306	0
	med	61.67858	55.22468	15.42187	10.88657	0	0	0.005871	154.6214	0
F_10_	avg	3.8134	2.6304	3.5438 × 10^−9^	0.2691	8.6234 × 10^−13^	3.91 × 10^−15^	8.6247 × 10^−13^	8.6291 × 10^−11^	8.88× 10^−16^
	std	6.8972 × 10^−15^	6.9631 × 10^−15^	2.7054 × 10^−24^	6.4129 × 10^−14^	5.6719 × 10^−28^	7.01 × 10^−30^	1.6240 × 10^−12^	5.3014 × 10^−11^	0
	bsf	2.757203	1.155151	2.64 × 10^−09^	0.156305	1.51 × 10^−14^	8.88 × 10^−16^	8.14 × 10^−15^	1.68 × 10^−18^	8.88 × 10^−16^
	med	3.120322	2.170083	3.64 × 10^−09^	0.261541	1.51 × 10^−14^	4.44 × 10^−15^	1.1 × 10^−13^	1.05 × 10^−11^	8.88 × 10^−16^
F_11_	avg	1.1973	0.0364	3.9123	0.5912	0.0013	2.03 × 10^−4^	5.3614 × 10^−7^	0	0
	std	4.8521 × 10^−15^	2.6398 × 10^−17^	4.0306 × 10^−14^	6.2914 × 10^−15^	6.1294 × 10^−17^	1.82 × 10^−17^	6.3195 × 10^−7^	0	0
	bsf	1.140471	7.29 × 10^−09^	1.519288	0.310117	0	0	4.23 × 10^−15^	0	0
	med	1.227231	0.029473	3.424268	0.582026	0	0	8.77 × 10^−07^	0	0
F_12_	avg	0.0469	0.4792	0.0341	0.0219	0.0364	0.007728	0.0372	0.0815	0.0583
	std	1.7456 × 10^−14^	9.3071 × 10^−15^	2.0918 × 10^−16^	2.6195 × 10^−14^	1.3604 × 10^−13^	8.07E-05	8.6391 × 10^−2^	0.0162	2.73 × 10^−16^
	bsf	0.018364	0.000145	5.57 × 10^−20^	0.002031	0.019294	0.001142	0.035428	0.077912	0.0452
	med	0.04179	0.1556	1.48 × 10^−19^	0.015181	0.032991	0.003887	0.050935	0.082108	0.1464
F_13_	avg	1.2106	0.5156	0.0017	0.3306	0.5561	0.193293	2.8041	0.4875	1.42866
	std	3.5630 × 10^−15^	4.1427 × 10^−16^	1.9741 × 10^−13^	5.6084 × 10^−15^	5.6219 × 10^−15^	0.022767	3.9514 × 10^−11^	0.1041	2.83× 10^−15^
	bsf	0.49809	9.99 × 10^−07^	1.18 × 10^−18^	0.038266	0.297822	0.029662	2.63175	0.280295	1.428663
	med	1.218053	0.043997	2.14 × 10^−18^	0.282764	0.578323	0.146503	2.66175	0.579854	2.976773

**Table 4:** Evaluation results of fixed-dimensional multimodal functions.

		GA	PSO	GSA	TLBO	GWO	WOA	TSA	MPA	POA
F_14_	avg	0.9969	2.3909	3.9505	2.4998	4.1140	1.106143	2.061	0.9980	0.9980
	std	6.3124 × 10^−14^	8.0126 × 10^−15^	8.9631 × 10^−15^	6.3014 × 10^−15^	1.3679 × 10^−14^	0.48689	5.6213 × 10^−7^	1.9082 × 10^−15^	0
	bsf	0.998004	0.998004	0.999508	0.998391	0.998004	0.998004	0.9979	0.9980	0.9980
	med	0.998018	0.998004	2.986658	2.275231	2.982105	0.998004	1.912608	0.9980	0.9980
F_15_	avg	0.0042	0.0528	0.0027	0.0031	0.0059	0.000463	0.0005	0.0028	0.0003
	std	1.6317 × 10^−17^	2.6159 × 10^−18^	3.6051 × 10^−18^	6.3195 × 10^−16^	3.0598 × 10^−17^	1.22 × 10^−7^	1.6230 × 10^−5^	1.2901 × 10^−14^	1.21× 10^−19^
	bsf	0.000775	0.000307	0.000805	0.002206	0.000307	0.000313	0.000264	0.00027	0.0003
	med	0.002074	0.000307	0.002311	0.003185	0.000308	0.000492	0.00039	0.0027	0.0003
F_16_	avg	−1.0307	−1.0312	−1.0309	−1.0310	−1.0316	−1.0316	−1.0314	−1.0315	−1.0316
	std	9.1449 × 10^−15^	3.2496 × 10^−15^	5.4162 × 10^−15^	1.3061 × 10^−14^	3.0816 × 10^−15^	2.38 × 10^−20^	6.0397 × 10^−15^	2.1679 × 10^−15^	1.93× 10^−18^
	bsf	−1.0316	−1.0316	−1.0316	−1.0316	−1.0316	−1.0316	−1.03161	−1.0316	−1.03163
	med	−1.0309	−1.0311	−1.0310	−1.0308	−1.0316	−1.0316	−1.0311	−1.0312	−1.03163
F_17_	avg	0.4401	0.7951	0.3980	0.3978	0.3981	0.39788	0.3987	0.3991	0.3978
	std	1.4109 × 10^−16^	3.9801 × 10^−5^	1.0291 × 10^−16^	2.1021 × 10^−15^	6.0391 × 10^−16^	1.42 × 10^−12^	6.1472 × 10^−15^	5.9317 × 10^−14^	0
	bsf	0.3978	0.3978	0.3978	0.3978	0.3978	0.397887	0.3980	0.3982	0.3978
	med	0.4016	0.6521	0.3979	0.3978	0.3979	0.397887	0.3990	0.3977	0.3978
F_18_	avg	4.3601	3.0010	3.0016	3.0010	3.0009	3.000009	3	3.0013	3
	std	2.6108 × 10^−15^	1.1041 × 10^−14^	3.7159 × 10^−15^	7.6013 × 10^−14^	5.0014 × 10^−14^	2.42 × 10^−15^	5.6148 × 10^−14^	2.3017 × 10^−14^	1.09× 10^−16^
	bsf	3.0002	3	3	3	3	3	3	3	3
	med	3.7581	3.0005	3.0008	3.0006	3.0006	3.000001	3	3.0009	3
F_19_	avg	−3.8519	−3.8627	−3.8627	−3.8615	−3.8617	−3.86068	−3.8205	−3.8627	−3.86278
	std	3.6015 × 10^−14^	7.0114 × 10^−14^	5.3419 × 10^−14^	1.0314 × 10^−14^	9.6041 × 10^−14^	6.55 × 10^−6^	6.7514 × 10^−14^	2.6197 × 10^−14^	6.45× 10^−16^
	bsf	−3.86278	−3.8627	−3.8627	−3.8625	−3.8627	−3.86278	−3.8366	−3.8627	−3.86278
	med	−3.8413	−3.8560	−3.8627	−3.8620	−3.8612	−3.86216	−3.8066	−3.8627	−3.86278
F_20_	avg	−2.8301	−3.2626	−3.0402	−3.1927	−3.2481	−3.22298	−3.3201	−3.3195	−3.3220
	std	3.7124 × 10^−15^	3.4567 × 10^−15^	5.2179 × 10^−13^	5.3140 × 10^−14^	3.3017 × 10^−14^	0.008173	6.5203 × 10^−14^	9.8160 × 10^−10^	1.97× 10^−16^
	bsf	−3.31342	−3.322	−3.322	−3.26174	−3.32199	−3.32198	−3.3212	−3.3213	−3.322
	med	−2.96828	−3.2160	−2.9014	−3.2076	−3.26248	−3.19935	−3.3206	−3.3211	−3.322
F_21_	avg	−4.2593	−5.4236	−5.2014	−9.2049	−9.6602	−8.87635	−5.1477	−9.9561	−10.1532
	std	2.3631 × 10^−8^	6.3014 × 10^−9^	5.8961 × 10^−8^	3.8715 × 10^−14^	5.3391 × 10^−14^	5.123359	6.1974 × 10^−12^	8.7195 × 10^−10^	1.93× 10^−16^
	bsf	−7.82781	−8.0267	−7.3506	−9.6638	−10.1532	−10.1531	−7.5020	−10.1532	−10.1532
	med	−4.16238	−5.10077	−3.64802	−9.1532	−10.1526	−10.1518	−5.5020	−10.1531	−10.1532
F_22_	avg	−5.1183	−7.6351	−9.0241	−10.0399	−10.4199	−9.33732	−5.0597	−10.2859	−10.4029
	std	6.1697 × 10^−14^	5.0610 × 10^−14^	5.0231 × 10^−11^	6.7925 × 10^−13^	6.1496 × 10^−14^	4.752577	3.1673 × 10^−14^	7.3596 × 10^−10^	3.57× 10^−16^
	bsf	−9.1106	−10.4024	−10.4026	−10.4023	−10.4021	−10.4028	−9.06249	−10.4029	−10.4029
	med	−5.0296	−10.4020	−10.4017	−10.1836	−10.4015	−10.4013	−5.06249	−10.4027	−10.4029
F_23_	avg	−6.5675	−6.1653	−8.9091	−9.2916	−10.1319	−9.45231	−10.3675	−10.1409	−10.5364
	std	5.6014 × 10^−14^	5.3917 × 10^−15^	8.0051 × 10^−14^	5.2673 × 10^−14^	2.6912 × 10^−15^	9.47 × 10^−9^	2.9637 × 10^−12^	5.0981 × 10^−10^	3.97× 10^−16^
	bsf	−10.2227	−10.5364	−10.5364	−10.5340	−10.5363	−10.5363	−10.3683	−10.5364	−10.5364
	med	−6.5629	−4.50554	−10.5360	−9.6717	−10.5361	−10.5349	−10.3613	−10.2159	−10.5364

**Table 5:** *p*-values obtained from Wilcoxon sum rank test.

Functions Type	Compared Algorithms							
	POA and MPA	POA and TSA	POA and WOA	POA and GWO	POA and TLBO	POA and GSA	POA and PSO	POA and GA
Unimodal	0.0156	0.0156	0.0156	0.0156	0.0156	0.0312	0.0156	0.0156
High-dimensional multimodal	0.3125	0.2187	0.1562	0.8437	0.3125	0.3125	0.1562	0.1562
Fixed-dimensional multimodal	0.0195	0.0039	0.0078	0.0117	0.0058	0.0195	0.0039	0.0019

**Table 6:** Sensitivity analysis of the POA to *N*.

Objective Function	Number of Population Members			
	20	30	50	80
F_1_	9.3343 × 10^−212^	1.6451 × 10^−235^	2.87 × 10^−258^	7.3038 × 10^−260^
F_2_	1.5489 × 10^−98^	2.303 × 10^−119^	1.42 × 10^−128^	2.0842 × 10^−132^
F_3_	1.6656 × 10^−206^	9.9891 × 10^−249^	1.879 × 10^−256^	2.1553 × 10^−259^
F_4_	6.0489 × 10^−112^	1.4332 × 10^−127^	2.36 × 10^−133^	3.6451 × 10^−136^
F_5_	28.4440	27.1418	27.1253	25.4195
F_6_	0	0	0	0
F_7_	0.0001	8.8865 × 10^−6^	9.37 × 10^−6^	1.3305 × 10^−6^
F_8_	−7727.8678	−8924.3072	−9336.7304	−9385.8725
F_9_	0	0	0	0
F_10_	8.88 × 10^−16^	8.88 × 10^−16^	8.88 × 10^−16^	8.88 × 10^−16^
F_11_	0	0	0	0
F_12_	0.2944	0.0369	0.0583	0.0142
F_13_	2.9548	2.0214	1.4286	2.0471
F_14_	1.6403	1.0120	0.9980	0.9980
F_15_	0.0024	0.0003	0.0003	0.0003
F_16_	−1.0311	−1.0314	−1.0316	−1.03163
F_17_	0.3987	0.3983	0.3978	0.3978
F_18_	3.0003	3.0001	3.0000	3.0000
F_19_	−3.8615	−3.8625	−3.8628	−3.8628
F_20_	−3.3041	−3.3120	−3.322	−3.322
F_21_	−7.3492	−10.1529	−10.1532	−10.1532
F_22_	−8.0110	−10.4023	−10.4029	−10.4029
F_23_	−8.6436	−10.5357	−10.5364	−10.5364

**Table 7:** Sensitivity analysis of the POA to *T*.

Objective Function	Maximum Number of Iterations			
	100	500	800	1000
F_1_	2.7725 × 10^−19^	6.2604 × 10^−115^	4.3539 × 10^−185^	2.87 × 10^−258^
F_2_	1.1541 × 10^−9^	3.5658 × 10^−57^	1.61505 × 10^−94^	1.42 × 10^−128^
F_3_	2.1172 × 10^−19^	5.0884 × 10^−117^	6.461 × 10^−180^	1.879 × 10^−256^
F_4_	5.9252 × 10^−10^	1.8962 × 10^−56^	3.1178 × 10^−92^	2.36 × 10^−133^
F_5_	28.9350	28.5274	28.3259	27.1253
F_6_	0	0	0	0
F_7_	0.0007	0.0001	9.0872 × 10^−5^	9.37 × 10^−6^
F_8_	−6753.5658	−8063.7455	−8208.3044	−9336.7304
F_9_	0	0	0	0
F_10_	1.1932 × 10^−16^	8.88 × 10^−16^	8.88 × 10^−16^	8.88 × 10^−16^
F_11_	0	0	0	0
F_12_	0.5768	0.2211	0.1673	0.0583
F_13_	2.8999	2.7595	2.7286	1.4286
F_14_	1.0012	0.9996	0.9980	0.9980
F_15_	0.0013	0.0007	0.0004	0.0003
F_16_	−1.0310	−1.0314	−1.0316	−1.03163
F_17_	0.3983	0.3972	0.3978	0.3978
F_18_	3.0172	3.0120	3.0001	3.0000
F_19_	−3.7928	−3.8598	−3.8628	−3.8628
F_20_	−3.2810	−3.3160	−3.3041	−3.322
F_21_	−9.8968	−9.6433	−9.8982	−10.1532
F_22_	−10.4002	−10.4018	−10.4022	−10.4029
F_23_	−10.5358	−10.5361	−10.5363	−10.5364

**Table 8:** Sensitivity analysis of the POA to *R*.

OF	*R* Value									
	0.1	0.2	0.3	0.4	0.5	0.6	0.7	0.8	0.9	1
F_1_	4.84 × 10^−244^	2.87 × 10^−258^	7.98 × 10^−246^	3.79 × 10^−244^	6.25 × 10^−240^	6.31 × 10^−235^	2.32 × 10^−231^	4.98 × 10^−227^	6.44 × 10^−224^	1.04 × 10^−221^
F_2_	1.50 × 10^−126^	1.42 × 10^−128^	2.72 × 10^−125^	7.70 × 10^−125^	2.01 × 10^−123^	3.85 × 10^−122^	1.89 × 10^−121^	2.56 × 10^−120^	4.69 × 10^−119^	6.50 × 10^−115^
F_3_	6.84 × 10^−256^	1.879 × 10^−256^	3.92 × 10^−251^	4.90 × 10^−248^	1.83 × 10^−244^	4.39 × 10^−241^	8.56 × 10^−236^	2.83 × 10^−236^	8.20 × 10^−235^	1.96 × 10^−234^
F_4_	3.50 × 10^−126^	2.36 × 10^−133^	8.99 × 10^−120^	1.96 × 10^−123^	1.90 × 10^−126^	2.60 × 10^−122^	4.96 × 10^−115^	4.04 × 10^−112^	1.40 × 10^−112^	6.74 × 10^−110^
F_5_	27.5583	27.1253	27.5641	27.5912	27.8162	28.4294	28.5964	28.6237	28.6907	28.7015
F_6_	0	0	0	0	0	0	0	0	0	0
F_7_	3.43 × 10^−5^	9.37 × 10^−6^	4.86 × 10^−5^	7.62 × 10^−5^	4.31 × 10^−5^	2.06 × 10^−4^	2.71 × 10^−4^	4.63 × 10^−4^	3.66 × 10^−4^	5.70 × 10^−4^
F_8_	−8934.1836	−9336.7304	−8963.8127	−8898.2760	−8702.3872	−8629.6948	−8485.2713	−8212.2289	−8070.2688	−7919.3914
F_9_	0	0	0	0	0	0	0	0	0	0
F_10_	8.88 × 10^−16^	8.88 × 10^−16^	8.88 × 10^−16^	8.88 × 10^−16^	8.88 × 10^−16^	8.88 × 10^−16^	8.88 × 10^−16^	8.88 × 10^−16^	8.88 × 10^−16^	8.88 × 10^−16^
F_11_	0	0	0	0	0	0	0	0	0	0
F_12_	0.1542	0.0583	0.0629	0.0701	0.0821	0.08659	0.08826	0.09184	0.09633	0.097571
F_13_	2.8516	1.4286	2.1295	2.5203	2.591	2.6314	2.4736	2.3871	2.7630	2.8532
F_14_	0.9980	0.9980	0.9980	0.9980	0.9980	0.9980	0.9980	0.9980	0.9980	0.9980
F_15_	0.0003	0.0003	0.0003	0.0003	0.0003	0.0003	0.0003	0.0003	0.0003	0.0003
F_16_	−1.03163	−1.03163	−1.03163	−1.03163	−1.03163	−1.03163	−1.03163	−1.03163	−1.03163	−1.03163
F_17_	0.3978	0.3978	0.3978	0.3978	0.3978	0.3978	0.3978	0.3978	0.3978	0.3978
F_18_	3.0000	3.0000	3.0000	3.0000	3.0000	3.0000	3.0000	3.0000	3.0000	3.0000
F_19_	−3.8628	−3.8628	−3.8628	−3.8628	−3.8628	−3.8628	−3.8628	−3.8628	−3.8628	−3.8628
F_20_	−3.322	−3.322	−3.322	−3.3219	−3.3218	−3.3218	−3.1984	−3.1821	−3.1167	−3.0126
F_21_	−10.1532	−10.1532	−10.1531	−10.1531	−10.1529	−10.1527	−9.8965	−9.9623	−9.2196	−9.1637
F_22_	−10.4029	−10.4029	−10.4027	−10.4027	−10.3827	−10.3561	−10.0032	−9.7304	−9.1931	−9.0157
F_23_	−10.5364	−10.5364	−10.5363	−10.5363	−10.2195	−10.0412	−9.6318	−9.2305	−9.1027	−10.0081

**Table 9:** Comparison results for pressure vessel design problem.

Algorithm		Optimum Variables			Optimum Cost
	*T_s_*	*T_h_*	*R*	*L*	
POA	0.778035	0.384607	40.31261	199.9972	5883.0278
MPA	0.782101	0.386813	40.51662	200	5915.005
TSA	0.78293	0.386583	40.52943	200	5918.816
WOA	0.782856	0.386606	40.52252	200	5920.845
GWO	0.849948	0.420657	44.03535	157.1635	6041.572
TLBO	0.821665	0.420022	41.95814	184.4906	6168.059
GSA	1.091229	0.954362	49.59196	170.3348	11608.05
PSO	0.756124	0.401538	40.65478	198.9927	5919.78
GA	1.105021	0.911112	44.67868	180.5572	6582.773

**Table 10:** Statistical results for a pressure vessel design problem.

Algorithm	Best	Mean	Worst	Std. Dev.	Median
POA	5883.0278	5887.082	5894.256	24.35317	5886.457
MPA	5915.005	5890.388	5895.267	2.894447	5889.171
TSA	5918.816	5894.47	5897.571	13.91696	5893.595
WOA	5920.845	6534.769	7398.285	534.3861	6419.322
GWO	6041.572	6480.544	7254.542	327.1705	6400.679
TLBO	6168.059	6329.924	6515.61	126.6723	6321.477
GSA	11608.05	6843.963	7162.87	5793.52	6841.052
PSO	5919.78	6267.137	7009.253	496.3761	6115.746
GA	6582.773	6647.309	8009.442	657.8518	7589.802

**Table 11:** Comparison results for speed reducer design problem.

Algorithm				Optimum Variables				Optimum Cost
	*b*	*m*	*p*	*l*_1_	*l*_2_	*d*_1_	*d*_2_	
POA	3.5	0.7	17	7.3	7.8	3.350215	5.286683	2996.3482
MPA	3.503341	0.7	17	7.3	7.8	3.352946	5.291384	3000.05
TSA	3.508443	0.7	17	7.381059	7.815726	3.359526	5.289411	3002.789
WOA	3.501769	0.7	17	8.3	7.8	3.354088	5.289358	3007.266
GWO	3.510256	0.7	17	7.410236	7.816034	3.359752	5.28942	3004.429
TLBO	3.510509	0.7	17	7.3	7.8	3.462751	5.291858	3032.078
GSA	3.6018	0.7	17	8.3	7.8	3.371343	5.291869	3052.646
PSO	3.512008	0.7	17	8.35	7.8	3.363882	5.290367	3069.095
GA	3.521884	0.7	17	8.37	7.8	3.368653	5.291363	3030.517

**Table 12:** Statistical results for speed reducer design problem.

Algorithm	Best	Mean	Worst	Std. Dev.	Median
POA	2996.3482	2999.88	3001.491	1.782335	2998.715
MPA	3000.05	3002.04	3006.292	1.933476	3001.586
TSA	3002.789	3008.25	3011.159	5.84261	3006.923
WOA	3007.266	3107.736	3213.743	79.70181	3107.736
GWO	3004.429	3031.264	3063.407	13.02901	3029.453
TLBO	3032.078	3068.37	3107.263	18.08866	3068.061
GSA	3052.646	3172.87	3366.564	92.64666	3159.277
PSO	3069.095	3189.072	3315.85	17.13229	3200.746
GA	3030.517	3297.965	3622.361	57.06912	3291.288

**Table 13:** Comparison results for welded beam design problem.

Algorithm		Optimum Variables			Optimum Cost
	*h*	*l*	*T*	*b*	
POA	0.205719	3.470104	9.038353	0.205722	1.725021
MPA	0.205604	3.475541	9.037606	0.205852	1.726006
TSA	0.205719	3.476098	9.038771	0.20627	1.72734
WOA	0.19745	3.315724	10.000	0.201435	1.820759
GWO	0.205652	3.472797	9.042739	0.20575	1.725817
TLBO	0.204736	3.536998	9.006091	0.210067	1.759525
GSA	0.147127	5.491842	10.000	0.217769	2.173293
PSO	0.164204	4.033348	10.000	0.223692	1.874346
GA	0.206528	3.636599	10.000	0.20329	1.836617

**Table 14:** Statistical results for welded beam design problem.

Algorithm	Best	Mean	Worst	Std. Dev.	Median
POA	1.724968	1.726504	1.728593	0.004328	1.725779
MPA	1.726006	1.727209	1.727445	0.000287	1.727168
TSA	1.72734	1.72851	1.728946	0.001158	1.728469
WOA	1.820759	2.232094	3.05067	0.324785	2.246459
GWO	1.725817	1.731064	1.743044	0.00487	1.728802
TLBO	1.759525	1.819111	1.874907	0.027565	1.821584
GSA	2.173293	2.546274	3.00606	0.256064	2.49711
PSO	1.874346	2.120935	2.321981	0.034848	2.098726
GA	1.836617	1.364618	2.036875	0.139597	1.937297

**Table 15:** Comparison results for tension/compression spring design problem.

Algorithm		Optimum Variables		Optimum Cost
	*d*	*D*	*p*	
POA	0.051892	0.361608	11.00793	0.012666
MPA	0.051154	0.34382	12.09792	0.012677
TSA	0.050188	0.341609	12.0759	0.012681
WOA	0.05001	0.310476	15.003	0.013195
GWO	0.05001	0.316019	14.22908	0.012819
TLBO	0.05079	0.334846	12.72523	0.012712
GSA	0.05001	0.317375	14.23152	0.012876
PSO	0.05011	0.310173	14.0028	0.013039
GA	0.05026	0.316414	15.24265	0.012779

**Table 16:** Statistical results for tension/compression spring design problem.

Algorithm	Best	Mean	Worst	Std. Dev.	Median
POA	0.012666	0.012688	0.012677	0.001022	0.012685
MPA	0.012677	0.012693	0.012724	0.005623	0.012696
TSA	0.012681	0.012706	0.01273	0.004157	0.012709
WOA	0.013195	0.014828	0.017875	0.002274	0.013202
GWO	0.012819	0.014474	0.017852	0.001623	0.014031
TLBO	0.012712	0.012849	0.013008	7.81E-05	0.012854
GSA	0.012876	0.013448	0.014222	0.000287	0.013377
PSO	0.013039	0.014046	0.016263	0.002074	0.013011
GA	0.012779	0.013079	0.015225	0.000375	0.012961

## Data Availability

Not applicable.

## Appendix A

The information of the objective functions used in the simulation section is presented in Table A1, Table A2 and Table A3.

**Table A1:** Unimodal functions.

Objective Function	Range	Dimensions	$F_{min}$
$F_{1}\left(x\right)=\overset{m}{\underset{i=1}{\sum }}x_{i}^{2}$	[−100, 100]	30	0
$F_{2}\left(x\right)=\overset{m}{\underset{i=1}{\sum }}\left|x_{i}\right|+\overset{m}{\underset{i=1}{\prod }}\left|x_{i}\right|$	[−10, 10]	30	0
$F_{3}\left(x\right)=\overset{m}{\underset{i=1}{\sum }}{\left(\overset{i}{\underset{j=1}{\sum }}x_{i}\right)}^{2}$	[−100, 100]	30	0
$F_{4}\left(x\right)=max\left\{\left|x_{i}\right|,\;\;1\leq i\leq m\;\right\}$	[−100, 100]	30	0
$F_{5}\left(x\right)=\overset{m-1}{\underset{i=1}{\sum }}\left[100{\left(x_{i+1}-x_{i}^{2}\right)}^{2}+{\left(x_{i}-1\right)}^{2})\right]$	[−30, 30]	30	0
$F_{6}\left(x\right)=\overset{m}{\underset{i=1}{\sum }}{\left(\left[x_{i}+0.5\right]\right)}^{2}$	[−100, 100]	30	0
$F_{7}\left(x\right)=\overset{m}{\underset{i=1}{\sum }}ix_{i}^{4}+random\left(0,1\right)$	[−1.28, 1.28]	30	0

**Table A2:** High-dimensional multimodal functions.

Objective Function	Range	Dimensions	F_min_
$F_{8}\left(x\right)=\overset{m}{\underset{i=1}{\sum }}-x_{i}\;\sin\left(\sqrt{\left|x_{i}\right|}\right)$	[−500, 500]	30	−12,569
$F_{9}\left(x\right)=\overset{m}{\underset{i=1}{\sum }}\left[\;x_{i}^{2}-10\cos\left(2\pi x_{i}\right)+10\right]$	[−5.12, 5.12]	30	0
$F_{10}\left(x\right)=-20\exp\left(-0.2\sqrt{\frac{1}{m}\overset{m}{\underset{i=1}{\sum }}x_{i}^{2}}\right)-\exp\left(\frac{1}{m}\overset{m}{\underset{i=1}{\sum }}\cos\left(2\pi x_{i}\right)\right)+20+e$	[−32, 32]	30	0
$F_{11}\left(x\right)=\frac{1}{4000}\overset{m}{\underset{i=1}{\sum }}x_{i}^{2}-\overset{m}{\underset{i=1}{\prod }}\cos\left(\frac{x_{i}}{\sqrt{i}}\right)+1$	[−600, 600]	30	0
$F_{12}\left(x\right)=\frac{\pi }{m}\;\left\{10\sin\left(\pi y_{1}\right)+\overset{m}{\underset{i=1}{\sum }}{\left(y_{i}-1\right)}^{2}\left[1+10\sin^{2}\left(\pi y_{i+1}\right)\right]+{\left(y_{n}-1\right)}^{2}\right\}+\overset{m}{\underset{i=1}{\sum }}u\left(x_{i},10,100,4\right),$ where $u\left(x_{i},a,i,n\right)=\{\begin{array}{c} k{\left(x_{i}-a\right)}^{n},\;\;\;\;\;\;\;\;\;\;\;\;\;\;\;x_{i}>-a;\\ 0,\;\;\;\;\;\;\;\;\;\;\;\;\;\;\;\;\;\;\;-a\leq \;x_{i}\;\leq a\\ k{\left(-x_{i}-a\right)}^{n},\;\;\;\;\;\;\;\;\;\;\;x_{i}<-a. \end{array};$	[−50, 50]	30	0
$F_{13}\left(x\right)=0.1\left\{\;\sin^{2}\left(3\pi x_{1}\right)+\overset{m}{\underset{i=1}{\sum }}{\left(x_{i}-1\right)}^{2}\left[1+\sin^{2}\left(3\pi x_{i}+1\right)\right]+{\left(x_{n}-1\right)}^{2}\left[1+\sin^{2}\left(2\pi x_{m}\right)\right]\right\}+\overset{m}{\underset{i=1}{\sum }}u\left(x_{i},5,100,4\right),$	[−50, 50]	30	0

**Table A3:** Fixed-dimensional multimodal functions.

Objective Function	Range	Dimensions	F_min_
$F_{14}\left(x\right)={\left(\frac{1}{500}+\overset{25}{\underset{j=1}{\sum }}\frac{1}{j+\sum_{i=1}^{2}{\left(x_{i}-a_{ij}\right)}^{6}}\right)}^{-1}$	[−65.53, 65.53]	2	0.998
$F_{15}\left(x\right)=\overset{11}{\underset{i=1}{\sum }}{\left[a_{i}-\frac{x_{1}\left(b_{i}^{2}+b_{i}x_{2}\right)}{b_{i}^{2}+b_{i}x_{3}+x_{4}}\right]}^{2}$	[−5, 5]	4	0.00030
$F_{16}\left(x\right)=4x_{1}^{2}-2.1x_{1}^{4}+\frac{1}{3}x_{1}^{6}+x_{1}x_{2}-4x_{2}^{2}+4x_{2}^{4}$	[−5, 5]	2	−1.0316
$F_{17}\left(x\right)={\left(x_{2}-\frac{5.1}{4\pi^{2}}x_{1}^{2}+\frac{5}{\pi }x_{1}-6\right)}^{2}+10\left(1-\frac{1}{8\pi }\right)\cos x_{1}+10$	[-5, 10] × [0, 15]	2	0.398
$F_{18}\left(x\right)=\left[1+{\left(x_{1}+x_{2}+1\right)}^{2}\left(19-14x_{1}+3x_{1}^{2}-14x_{2}+6x_{1}x_{2}+3x_{2}^{2}\right)\right]\cdot \left[30+{\left(2x_{1}-3x_{2}\right)}^{2}\cdot \left(18-32x_{1}+12x_{1}^{2}+48x_{2}-36x_{1}x_{2}+27x_{2}^{2}\right)\right]$	[−5, 5]	2	3
$F_{19}\left(x\right)=-\overset{4}{\underset{i=1}{\sum }}c_{i}\;\exp\left(-\overset{3}{\underset{j=1}{\sum }}a_{ij}{\left(x_{j}-P_{ij}\right)}^{2}\right)$	[0, 1]	3	−3.86
$F_{20}\left(x\right)=-\overset{4}{\underset{i=1}{\sum }}c_{i}\;\exp\left(-\overset{6}{\underset{j=1}{\sum }}a_{ij}{\left(x_{j}-P_{ij}\right)}^{2}\right)$	[0, 1]	6	−3.22
$F_{21}\left(x\right)=-\overset{5}{\underset{i=1}{\sum }}{\left[\left(X-a_{i}\right){\left(X-a_{i}\right)}^{T}+6c_{i}\right]}^{-1}$	[0, 10]	4	−10.1532
$F_{22}\left(x\right)=-\overset{7}{\underset{i=1}{\sum }}{\left[\left(X-a_{i}\right){\left(X-a_{i}\right)}^{T}+6c_{i}\right]}^{-1}$	[0, 10]	4	−10.4029
$F_{23}\left(x\right)=-\overset{10}{\underset{i=1}{\sum }}{\left[\left(X-a_{i}\right){\left(X-a_{i}\right)}^{T}+6c_{i}\right]}^{-1}$	[0, 10]	4	−10.5364
